# A 4-bp deletion in the 5’UTR of *TaAFP-B* is associated with seed dormancy in common wheat (*Triticum aestivum* L.)

**DOI:** 10.1186/s12870-019-1950-4

**Published:** 2019-08-09

**Authors:** Yumei Feng, Meng Liu, Zeng Wang, Xianlin Zhao, Bing Han, Yanping Xing, Maoyan Wang, Yan Yang

**Affiliations:** 1 College of Life Sciences, Inner Mongolia Agricultural University, Inner Mongolia Key Laboratory of Plant Stress Physiology and Molecular Biology, Erdos Road, Hohhot, 010018 Inner Mongolia China; 2 Wheat Research Institute, Henan Academy of Agricultural Sciences, Henan Key Laboratory of Wheat Biology, National Engineering Laboratory for Wheat, Key Laboratory of Wheat Biology and Genetic Breeding in Central Huang-Huai Region, Ministry of Agriculture, Zhengzhou, 450002 China

**Keywords:** Wheat, Seeds dormancy, TaAFPs, Marker, Transcription, Translation

## Abstract

**Background:**

AFP is a negative regulator of ABA signaling that promotes ABI5 protein degradation and weakens regulation of ABA signaling by targeting upstream genes of *ABI5*, and *TaABI5* gene was seed-specific, and accumulated during wheat grain maturation and dormancy acquisition, which played an important role in seed dormancy; *TaAFP* has a conserved domain with *AFP*, so *TaAFP* may also play an important role in seed dormancy in wheat.

**Results:**

Two allelic variants of *TaAFP* were identified on chromosome 2BS in common wheat, and designated as *TaAFP-B1a* and *TaAFP-B1b*. Sequence analysis showed a 4-bp deletion in the 5’UTR region of *TaAFP-B1b* compared with *TaAFP-B1a*. Based on the 4-bp deletion, a co-dominant functional marker of *TaAFP-B* was developed and designated as AFPB. The genotype generating a 203-bp fragment (*TaAFP-B1b*) was more resistant to pre-harvest sprouting than the genotype producing a 207-bp fragment (*TaAFP-B1a*) in a test of 91 white-grained Chinese wheat cultivars and advanced lines. The average germination index(GI) values of *TaAFP-B1a* and that of *TaAFP-B1b* were 45.18 and 30.72%, respectively, indicating a significant difference (*P* < 0.001). Moreover, the 4-bp deletion located in the 5’UTR not only affected the transcription level of *TaAFP-B* but also affected the mRNA decay, reduced the translation level of *GUS* and *tdTomatoER* and GUS activity in wheat leaves of transient expression. The transcript expression and the mRNA half-life value of *TaAFP-B1a* in developing seeds and mature seeds were much higher than those of *TaAFP-B1b*.

**Conclusion:**

We identified a 4-bp InDel in the 5’UTR of *TaAFP-B*, which affected the mRNA transcription level, mRNA decay, translation levels of *GUS* and *tdTomatoER*, GUS activity, and was significantly associated with seed dormancy in common wheat. A functional marker was developed and validated based on this InDel.

**Electronic supplementary material:**

The online version of this article (10.1186/s12870-019-1950-4) contains supplementary material, which is available to authorized users.

## Background

A high level of seed dormancy plays a pivotal role in resistance to pre-harvest sprouting (PHS), providing a mechanism for plants to delay germination until conditions are optimal for survival of the next generation [1, 2]. The balance of abscisic acid (ABA) and gibberellin (GA) levels and sensitivity is a major regulator of dormancy status. The mechanism of ABA sensitivity in seeds has been extensively studied in Arabidopsis. Some genes associated with seed dormancy have been identified as factors in the ABA signaling and ABA synthesis pathway [3–6]. *abi5* is insensitive to an ABA-induced post-germination growth arrest [7], and alters activity of an ABA-inducible late embryo genesis abundant (LEA) gene promoter [8]. *ABI5* is mainly expressed in dry seeds, and its expression significantly decreases after germination [9, 10]. Expression of *ABI5* is induced strongly by exogenous ABA [4], and is regulated by *ABI3*, *HYL1* and *HY5* [11, 12], but is repressed by WRKY2, WRKY40, WRKY18 and WRKY60 [13, 14]. Both *abi3* and *abi5* mutants were initially recovered by virtue of their ability to germinate in the presence of ABA [7, 15, 16]. The Arabidopsis *ABI3* gene is an orthologs of *Vp-1* and is required for appropriate *ABI5* expression [11, 10, 17]. *ABI3* encodes a transcription factor and acts together with ABI5 to govern embryonic gene expression and seed sensitivity to ABA [7, 15, 16].

ABI5 binding protein (AFP), a novel negative regulator of ABA signaling that works by facilitating the degradation of ABI5 [18], was isolated using yeast two-hybrid assays; AFP functions in developing seeds and young seedlings [18]. AFP gene transcription and translation increased during seed development and desiccation, ultimately reaching plateau values in mature seeds [18]. *ABI5* acts as a critical factor in maturation, dormancy development of seeds, or the dehydration tolerance of young seedlings of Arabidopsis [19, 20].

In wheat (*Tritivum aestivum*), many genes or QTL are associated with tolerance to PHS, and molecular markers have been developed based on these genes and QTL [21–26]. Several major QTL [27–29] associated with PHS tolerance were found and some genes associated with PHS tolerance were cloned [21, 30–37]. *TaABI5* genes one of which is the same as *TaABF*, and *TaABF* mRNA was seed-specific and accumulated during wheat grain maturation and dormancy acquisition, which played an important role in seed dormancy were isolated in wheat [32]. *TaABI5s* were expressed in developing grain, roots, and leaves [38]. Three wheat *AFP* genes (*TaAFPs*) were isolated, located on the short arms of chromosomes 2A, 2B, and 2D, and designated *TaAFP-A*, *TaAFP-B,* and *TaAFP-D*, respectively [38]. The structure of *TaAFP* consisted of one intron and two exons, including a nuclear localization domain (119–133 aa of *AtAFP*) in the middle of the deduced amino acid sequence and an ABI5-binding domain (284–335 aa of *AtAFP*) in the C-terminal region.

*ABI5* has an important function in maturation and dormancy development of seeds in Arabidopsis, while AFP promotes ABI5 protein degradation [18]. Moreover, TaAFP has a conserved domain (60 and 69%) with AtAFP in the region of nuclear localization and the ABI5 binding domain, respectively [38]. Therefore, *TaAFP* may play an important role in seed dormancy in wheat. The objectives of the present study were to: (1) identify allelic variations at the *TaAFP* locus in Chinese wheat varieties with different levels of seed dormancy; (2) develop a functional marker for use in marker-assisted selection for PHS tolerance; and (3) characterize the transcription regulation mechanism of *TaAFP*. The identification of new alleles of *TaAFP* associated with different seed dormancy could also contribute to our understanding of the mechanisms underlying seed dormancy or PHS tolerance in common wheat.

## Results

### Sequence analysis of three *TaAFP* homologs in varieties with different levels of seed dormancy

Full sequences of *TaAFP-A*, *TaAFP-B,* and *TaAFP-D* were cloned using genome-specific primers (Table 1). Six new alleles of *TaAFPs* were found and named according to the 2005 Supplement of the Wheat Gene Catalogue [39]. In these germplasm, *TaAFP-A1a* and *TaAFP-A1b* were on chromosome 2AS; *TaAFP-B1a* and *TaAFP-B1b* were on chromosome 2BS; and *TaAFP-D1a* and *TaAFP-D1b* were on chromosome 2DS. *TaAFP-A* was amplified and sequenced with three primer sets TaAFP-AF1/R1, TaAFP-AF2/R2, and TaAFP-AF3/R3 from 10 varieties mentioned above. Two new alleles of *TaAFP-A* were found and designated *TaAFP-A1a* and *TaAFP-A1b*.

**Table 1:** The primer sets used in this study

Primer Set	Upstream (5′-3′)	Downstream (5′-3′)	Primer Anneal Temperature (°C)	Fragment Size (bp)
TaAFP-AF1/R1	GATTCTACTGGCTCTGCTT	GAGCAAGGAAGCGCAATAG	57	808
TaAFP-AF2/R2	CCGTCGTCGACCAGGGGAA	CAGACTCTGACGCTACGTAACTC	63	568
TaAFP-AF3/R3	GAGATGCCTTCTACGGCATG	GAGGAAGTTGCCGTGGCAA	60	613
TaAFP-BF1/R1	GATTCTGCTGGCTCTGCTT	CAAGGGAACGCAATAGAAC	57	828
TaAFP-BF2/R2	CCGTCGTCGACCAGGGGAA	ACCCTGACGCTACGTAACTGT	62	587
TaAFP-BF3/R3	GAGATGCCTTCTACGGCATG	CCCCCTAGGTACATTCCGA	59	733
TaAFP-DF1/R1	GATTGTGCTGGCTCTGCTT	TTGGAGTAGCAGGGAAGCT	57	770
TaAFP-DF2/R2	CCGTCGTCGACCAGGGGAA	TCCCTCCGTTCCAAAATAGATGACT	62	618
TaAFP-DF3/R3	GAGATGCCTTCTACGGCATG	ACGATGTGCCTGAGCGGA	60	706
AFPBF/R	CTTCCTGAGAATTTGGCCGT	TGAGCTCGACCACCTCGTCG	61	207,203
ACTINF/R	GTTTCCTGGAATTGCTGATCGCAT	CATTATTTCATACAGCAGGCAAGC	62	410
Q-TaAFP-BF/R	ACCTCCTCAAGCATGCCGG	ACTTGTTCTGGTTGCTGGCA	66	102
Q-TaAFP-AF/R	CCCTCCTCAAGCATGCCCG	GCTGGCATTG TTATTATTATCG	59	100
Q-TaAFP-DF/R	GCTTCCTCAAGCATGCCGGA	CGTCCACCTTGGAGGAGACT	56	289
Q-ABI5F/R	GGAAGAAGTCACCTCRCACC	GAGGCAAGGAGAACGACT	62	310
Pro F/R	GATTCTGCTGGCTCTGCTT	ACGGCCAAATTCTCAGGAAG	59	152

Compared with the *TaAFP-A* (AB360911) [38], 6 SNPs were found in the full sequence of *TaAFP-A1a*: an A to G transversion was observed at − 197 bp in the 5’UTR; 2 SNPs were located in exons (i.e. C to T at position 94 bp in the first exon, and G to A at position 1523 bp in the second exon) that cause changes of amino acids Gla to Val and Glu to Lys, respectively); and the other 3 SNPs (i.e. G to T, T to A, and G to A) were present in the introns at positions of 529, 717, and 980 bp, respectively. For *TaAFP-A1b*, 4 SNPs were found: a C to T transversion at 94 bp in the first exon causeing a change of amino acid from Gla to Val; and the other 3 SNPs (i.e. T to A, C to A, and C to A) were located in the introns at positions of 717, 764, and 976 bp, respectively (Additional file 1: Figure S1).

*TaAFP-B* was amplified with the primer sets TaAFP-BF1/R1, TaAFP-BF2/R2, and TaAFP-BF3/R3. Two alleles of *TaAFP-B* were found, designated *TaAFP-B1a* and *TaAFP-B1b*. Compared with the *TaAFP-B* gene (GenBank accession AB360912) [38], *TaAFP-B1a* had 8 SNPs and 2 insertions. Four SNPs (i.e. G to A, G to A, T to C, and G to T at positions − 199 bp, − 152 bp, − 27 bp, and − 26 bp, respectively) were found in the 5’UTR; 2 SNPs (i.e. A to G at position 203 bp that causes change of amino acid from Tyr to Cys, and T to C at 1238 bp that is a synonymous mutation) were found in the first and second exon, respectively; 2 SNPs (i.e. T to C and G to A at position 574 bp and 1102 bp) were observed in introns; and the 2 insertions (G insertion and CT insertion at positions − 45 bp and − 25 bp) were present in the 5’UTR.

*TaAFP-B1b* had 3 SNPs, 1 insertion, and 1 deletion. One SNP was a C to T at position − 199 bp was found in the 5’UTR; another SNP was A to G at position 1252 bp that causes a change of amino acid from Glu to Gly; and the final SNP was T to C at position 574 bp was identified in an intron; there was a G insertion at position − 45 bp and CT deletion at position − 25 bp were found in the 5’UTR. In addition, a polymorphic fragment was detected in the 10 varieties with different levels of seed dormancy amplified with primer set TaAFP-BF1/R1. An 830-bp fragment was amplified in genotypes of *TaAFP-B1a*, whereas an 826-bp fragment was generated in genotype of *TaAFP-B1b*. Compared with the *TaAFP-B1a* genotype, a 4-bp deletion (CTCT) in the 5’UTR was present in *TaAFP-B1b* (Additional file 2: Figure S2).

The full sequence of *TaAFP-D* was amplified with the genome-specific primer sets TaAFP-DF1/R1, TaAFP-DF2/R2, and TaAFP-DF3/R3. Two new alleles of *TaAFP-D* were found and designated *TaAFP-D1a* and *TaAFP-D1b*. Compared with the *TaAFP-D* gene (GenBank accession AB360913) [38], 3 SNPs were found in *TaAFP-D1a*: 2 SNPs were located in the second exon (i.e. A to G transversions at positions 1502 and 1530 bp, resulting in changes of amino acids Ile to Met and Ser to Gly, respectively), and another SNP was located in the intron at 1273 bp. Five SNPs were found in the full sequence of *TaAFP-D1b*: 2 SNPs (i.e. A to G transition at positions 1502 and 1530 bp) were located in the second exon leading to change of amino acids Ile to Met and Ser to Gly, respectively); and the other 3 SNPs (i.e. G to A, T to C, and G to C) were detected in the intron at positions of 742, 995, and 1071 bp (Additional file 3: Figure S3).

### Development and validation of the STS marker AFPB for PHS tolerance

**Fig. 1:**
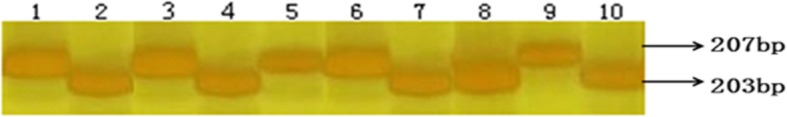
PCR fragments amplified with the primer set TaAFP-BF1/R1 from 10 wheat cultivars and lines. 1: Jimai 19 (*TaAFP-B1a*); 2: Jinan 16 (*TaAFP-B1b*); 3: Zhou 8425B (*TaAFP-B1a*); 4: Yangxiaomai (*TaAFP-B1b*); 5: Zhoumai 16 (*TaAFP-B1a*); 6: Yanfu 188 (*TaAFP-B1a*); 7: Langzhongbaimaizi (*TaAFP-B1b*); 8: Wanxianbaimaizi (*TaAFP-B1b*); 9: Yongchuanbaimaizi (*TaAFP-B1a*); 10: Xiaobaiyuhua (*TaAFP-B1b*)

Based on the sequence analysis, an STS marker of *TaAFP-B*, designated AFPB and located in the 5’UTR, was developed and used for association analysis with 91 Chinese varieties and advanced lines. Among the 91 varieties and lines tested, 23 possessed the allele *TaAFP-B1a* with a 207-bp fragment, whereas 68 had *TaAFP-B1b* with a 203-bp fragment (Additional file 4: Table S1 and Fig. 1). The GI values of the 91 varieties were consistent over the 2 years (r = 0.631, *P* < 0.01), with mean values and standard deviations of 0.345 ± 0.17 in 2006 and 0.343 ± 0.16 in 2007. Analysis of variance indicated significant differences (*P* < 0.001) between the two genotypes. The genotype *TaAFP-B1a* with a 207 bp fragment was more susceptible to PHS with an average GI value of 0.452, compared with *TaAFP-B1b* with an average GI value of 0.307, exhibiting a significant association of *TaAFP-B* with PHS tolerance.

### Expression of the *TaAFP-B* gene in Zhou 8425B and Wanxianbaimaizi at different developmental stages

**Fig. 2:**
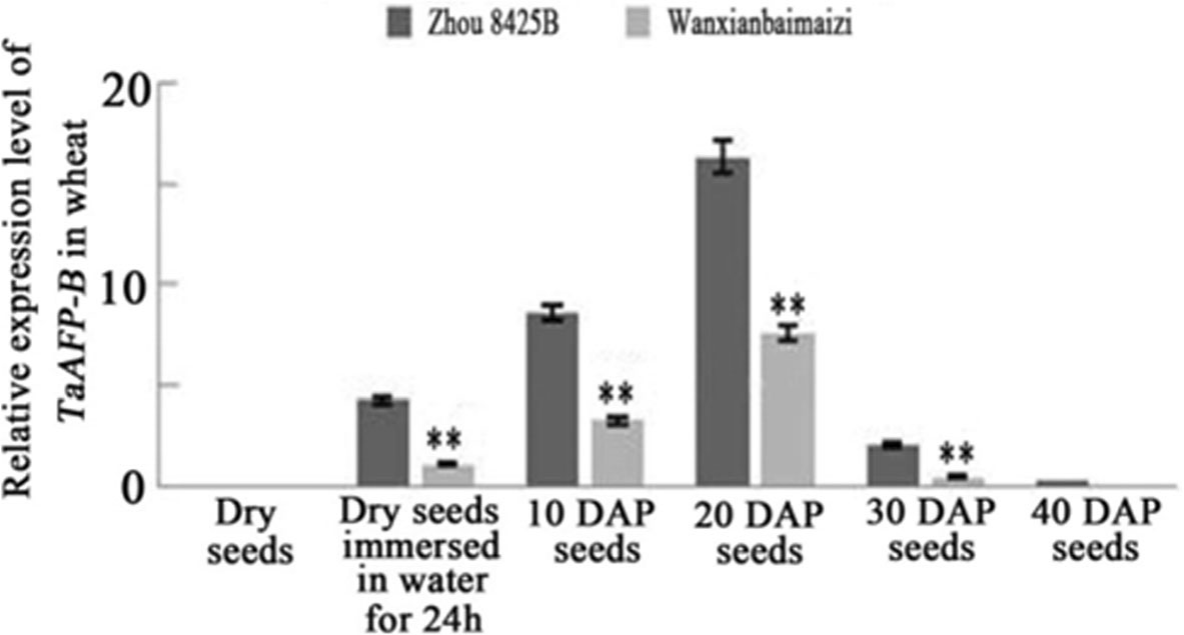
Transcript expression level of *TaAFP-B* with real time PCR in cultivars Zhou 8425B (*TaAFP-*B1a) and Wanxianbaimaizi (*TaAFP-B1b*). “**” means *p* < 0.01

To evaluate the potential influence of different alleles and characterize the expression patterns of *TaAFP-B1a* and *TaAFP-B1b* in different cultivars, the expression patterns of Zhou 8425B (*TaAFP-B1a*) and Wanxianbaimaizi (*TaAFP-B1b*) were determined using real-time Quantitative PCR (RT-qPCR) analysis. The transcript expression levels of *TaAFP-B1a* were higher than that of *TaAFP-B1b* in seeds at 10 days after pollination (DAP), 20 DAP, 30 DAP, 40 DAP, and dry mature seeds immersed in water for 24 h (Fig. 2). In addition, the transcript expression levels of *TaAFP-B1a* and *TaAFP-B1b* had a trend of first increasing and then decreasing in seeds of different developmental stages, reaching the highest level at 20 DAP and the lowest at 40 DAP (Fig. 2). No transcript expression of *TaAFP-B1a* and *TaAFP-B1b* was detected in dry seeds. The results illustrated that allelic variation in the 5’UTR of *TaAFP-B* affected the transcript expression level in seeds at 10 DAP, 20 DAP, 30 DAP, and 40 DAP and in dry mature seeds immersed in water for 24 h.

### Effect of the 4-bp deletion in the 5’UTR on the mRNA decay of *TaAFP-B*

**Fig. 3:**
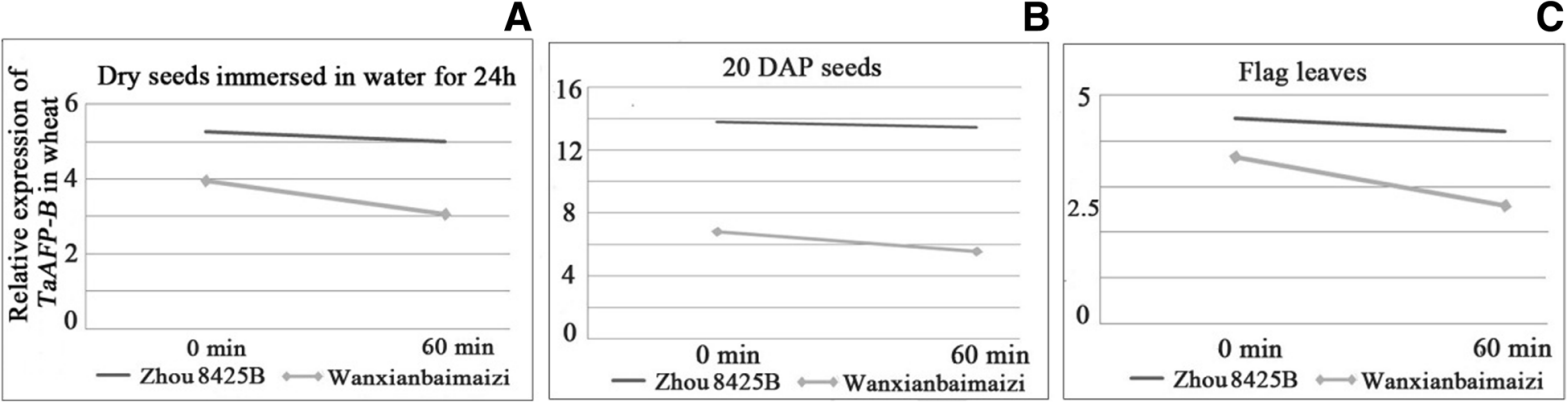
The mRNA expression level of genotypes of Zhou 8425B (*TaAFP-B1a*) and Wanxianbaimaizi (*TaAFP-B1b*) treated with Cordycepin for 0 min and 60 min in dry seeds immersed in water for 24 h, 20 DAP seeds, and flag leaves. **a** Dry seeds immersed in water for 24 h: the half-life of *TaAFP-B* is 154.00 min in Zhou 8425B and 46.20 min in Wanxianbaimaizi; **b** 20 DAP seeds: the half-life of *TaAFP-B* is 115.50 min in Zhou 8425B and 33.32 min in Wanxianbaimaizi; **c** Flag leaves: the half-life of *TaAFP-B* is 144.38 min in Zhou 8425B and 39.60 min in Wanxianbaimai

The results showed that *TaAFP-B* mRNA half-life (t_1/2_) of Zhou 8425B with the allele *TaAFP-B1a* was 144.38 min, 154.00 min, and 115.50 min, in flag leaves, dry mature seeds, and seeds at 20 DAP, respectively, whereas those of Wanxianbaimaizi were 39.60 min, 46.20 min, and 33.32 min, respectively (Fig. 3). This indicated that *TaAFP-B* mRNA of Zhou 8425B with *TaAFP-B1a* was more stable than that of Wanxianbaimaizi with *TaAFP-B1b*.

### The mRNA patterns of *TaAFPs* and *TaABI5* at different seed developmental stages

**Fig. 4:**
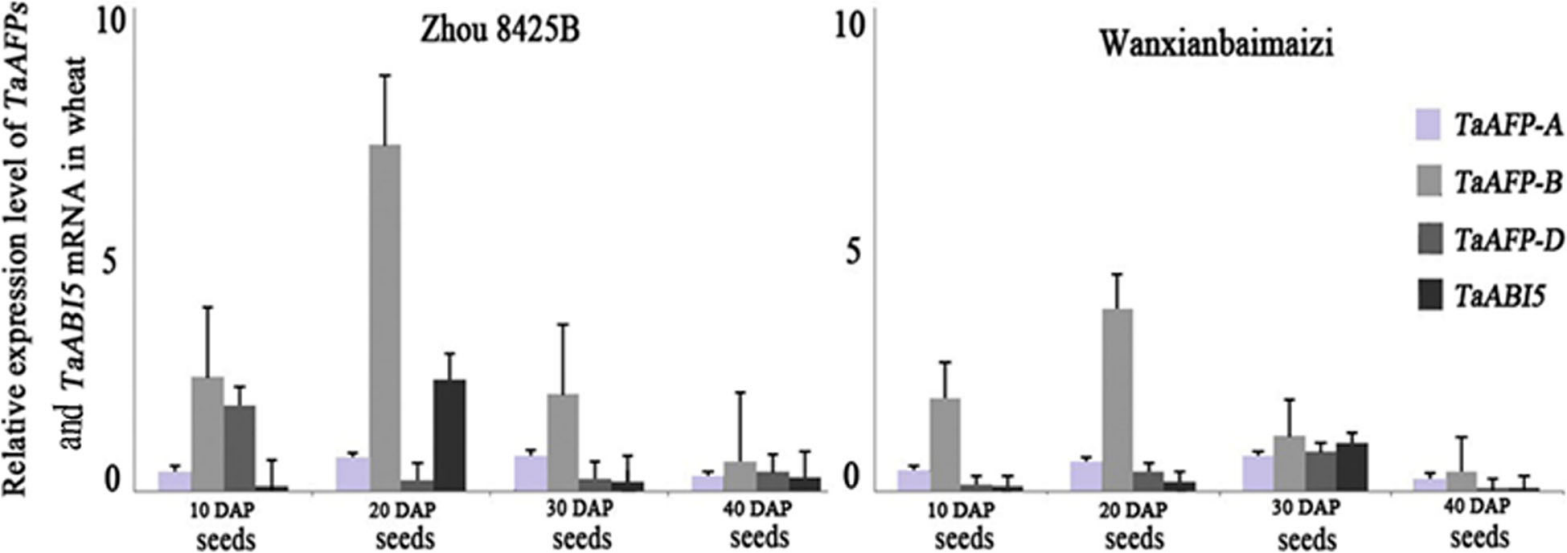
Expression pattern of *TaAFPs* and *TaABI5* in seeds at 10 DAP, 20 DAP, 30 DAP, and 40 DAP in Zhou 8425B and Wanxianbaimaizi using real time PCR

RT-qPCR showed that *TaAFP-B* had the highest transcription level of the four genes: *TaAFP-A*, *TaAFP-B*, *TaAFP-D,* and *TaABI5* at 10 DAP, 20 DAP, 30 DAP, and 40 DAP, respectively, in two genotypes of Zhou 8425B and Wanxianbaimaizi. Transcription levels of these four genes also had the general trend of gradually increasing and then decreasing during seed development. At the same time, *TaAFP-B* had a higher transcriptional level in Zhou 8425B than in Wanxianbaimaizi at each observation time point (Fig. 4). The most abundant *TaAFP-B* transcript levels in Zhou 8425B and Wanxianbaimaizi were detected at 20 DAP, while the highest transcriptional level of *TaABI5* occurred at 20 DAP in Zhou 8425B and at 30 DAP in Wanxianbaimaizi (Fig. 4). These results indicated that the time of the highest transcription level of *TaABI5* was latter than that of *TaAFP-B* in Wanxianbaimaizi; this might be another reason why Wanxianbaimaizi has a higher capacity of seed dormancy than Zhou 8425B.

### Transient expression of tdTomatoER and GUS-tagged pITV1-*TaAFP-Ba/bF* in wheat leaves

**Fig. 5:**
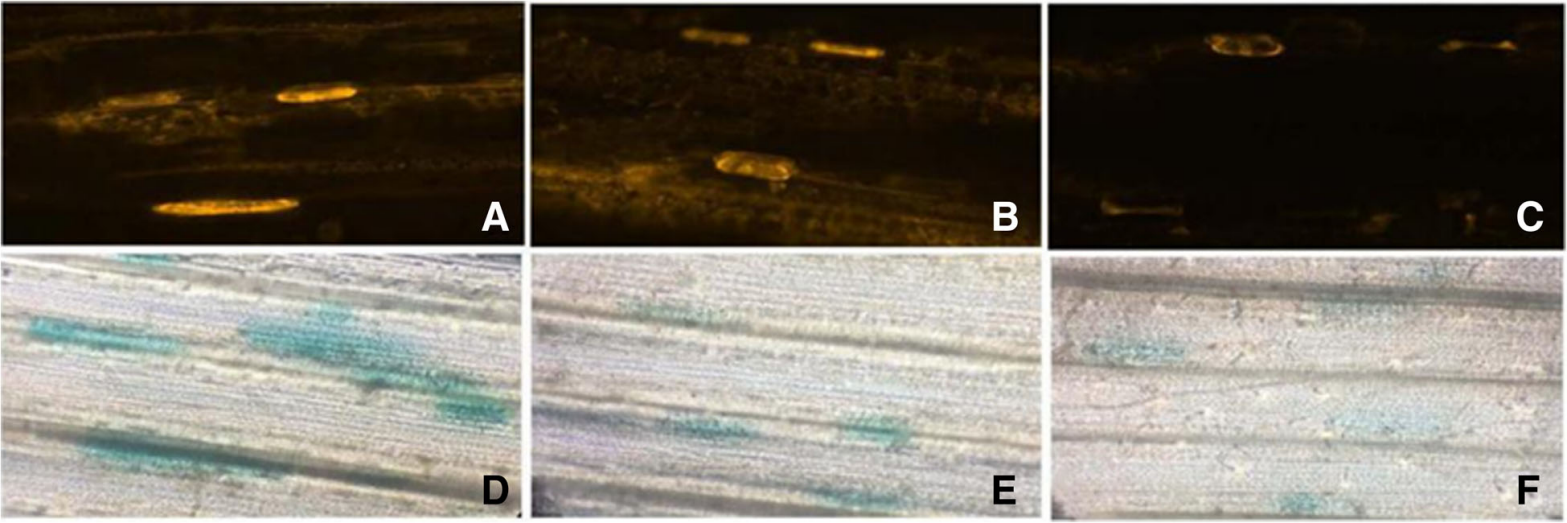
Transient experssion of tdTomatoER and GUS-Tagged *TaAFP-Ba/bF* in wheat leaves by confocal laser scanning microscope and optical microscope. **a** The expression of red fluorescence tdTomatoER in leaves of wheat by transforming *TaAFP-BaF* gene, Meanintensity: 5714.24, meandensity: 3.96; **b** The expression of red fluorescence tdTomatoER in leaves of wheat by transforming *TaAFP-BbF* gene, meanintensity: 101.22, meandensity: 3.43; **c** The expression of red fluorescence tdTomatoER in leaves of wheat in control, meanintensity: 43.14; meandensity: 3.12; **d** The expression of GUS in wheat leaves by transforming *TaAFP-BaF* gene; **e** The expression of GUS in wheat leaves by transforming *TaAFP-BbF* gene; **f** The expression of GUS in wheat leaves in control

The plant transient expression vectors pITV1-*TaAFP-BaF*, pITV1-*TaAFP-BbF*, and pITV1#1534 (control) were separately transformed into wheat leaves of Zhou 8425B using biolistic bombardment. The fluorescence intensity of *tdTomatoER* gene expression in leaves of wheat were observed with a confocal laser scanning microscope after the transformation for 12–18 h in a dark environment, then the *GUS* expression was observed with an optical microscope after treatment with dye and decoloring solution of GUS. The results showed that the fluorescence intensity of *tdTomatoER* gene expression and in the leaves was in the following order: pITV1-*TaAFP-BaF* > pITV1-*TaAFP-BbF* > pITV1#1534 (Fig. 5a, b, and c). In addition, *GUS* gene expression showed the same trend with the fluorescence intensity of *tdTomatoER* gene expression (Fig. 5: d, e, and f). These results indicated that the 4 bp deletion in the 5’UTR of *TaAFP-B* reduced the translation level of *tdTomatoER* and *GUS.*

### Quantitative analysis of *TaAFP-Ba/bF* activity

**Fig. 6:**
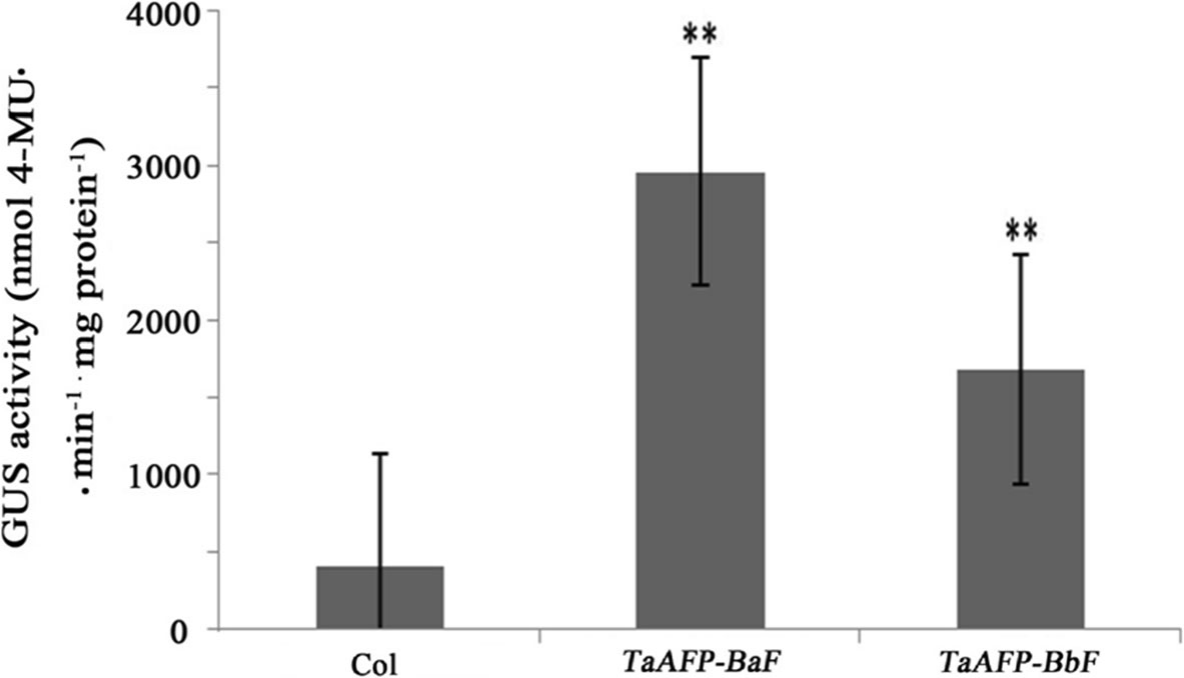
Quantitative analysis of *TaAFP-Ba/bF*::GUS transgenic leaves. “**”means *p* < 0.01

To investigate the quantitative of *TaAFP-Ba/bF* sequence of promoter activity, total proteins were extracted from wheat leaves of transient expression. The GUS activity was determined by fluorometric assays. The GUS expression level of *TaAFP-BaF::GUS* and *TaAFP-BbF::GUS* transient expression leaves were significantly highter than Col (*P* < 0.01)(Fig. 6). Otherwise, the GUS expression level and activity in *TaAFP-BaF::GUS* were higher than *TaAFP-BbF::GUS* (*P* < 0.01). The results indicated that the 4 bp deletion in the 5’UTR of *TaAFP-B* decreased the *GUS* activity.

## Discussion

The 800-bp region in the 5’UTR upstream from the start codon ATG of *TaAFPs* is relatively well conserved [38]. Nevertheless, in the present study, five allelic variants in the 5’UTR of *TaAFP-A* and *TaAFP-B* were found in 10 varieties (Additional file 1: Figure S1 and Additional file 2: Figure S2), indicating rich allelic variations in the 5’UTR of *TaAFP-A* and *TaAFP-B* in these germplasms. In addition, no alleles with the same to sequences as AB360911, AB360912, or AB360913 [38] were found in these 10 varieties, which may be attributed to the limited varieties sequenced. Furthermore, among the allelic variations present in *TaAFP-A*, *TaAFP-B* and *TaAFP-D* loci, only a CTCT deletion at the *TaAFP-B1b* locus was associated with seed dormancy in 91 varieties.

Analysis of *TaAFP* sequence showed some transcription factor binding elements in the region of 800 bp located upstream from the start code ATG, including ABA response elements ABRE, G-box, CACA, AACAA, Dof, RAVI, Myb-type transcription factor elements P, GAmyb, DRE/CRT and elements for cold and dehydration response [38]. Although the 4-bp deletion in the 5’UTR of *TaAFP-B1b* did not reside in any of the elements mentioned above, it changed the number of adjacent CT repeats from 9 to 7. Previous reports indicated that the number of repeat sequences in the promoter region of a gene could affect the expression level [40, 41]. Further study also showed that the 4-bp (CTCT) deletion changed the mRNA half-life and further transcription level (Figs. 2 and 3). The sequence with 9 CT repeats might increase the stability of mRNA and the expression level of *TaAFP-B1a*, which could enhance the binding of related transcription factors through changing the secondary structure of the upstream region of *TaAFP-B1a* during transcription.

Altering mRNA stability under some conditions plays an important role in the dynamic control of gene expression [42]; the rate of mRNA decay is an essential element of post-transcriptional regulation in all organisms, because the stability of mRNA determines how fast the equilibrium level of a new protein will be reached [43]. Thus, the half-life of mRNA will influence the stochastic fluctuation in the production rate of the corresponding protein [44]. mRNA transcripts are protected from degradation by exoribonuclease by 5′ capping and 3′ poly A structures [45]. In this study, compared with the sequence of *TaAFP-B1a*, a 4-bp deletion (CTCT) located at − 25 bp of the 5’UTR was found in *TaAFP-B1b*, and the mRNA half-life values of *TaAFP-B1b* and *TaAFP-B1a* were significantly different. More stable mRNA and a higher transcriptional level existed in *TaAFP-B1a* genotype.

The mRNA degradation data (Fig. 4) in this study suggest that the 4-bp deletion (CTCT) in the 5’UTR of *TaAFP-B1b* forms a “hot spot” for degradation by endogenous ribonucleases, whereas the region of *TaAFP-B1a* with “CTCT” sequence might be engineered to be more stable, leading to increased mRNA half-life. Consequently, more protein production and higher expression levels of tdTomatoER and GUS were observed in pITV1-*TaAFP-BaF* compared to pITV1-*TaAFP-BbF*, and higher GUS activity were detected in *TaAFP-BaF*::GUS transient expression wheat leaves than *TaAFP-BbF*::GUS, which indicated that the difference of the 4-bp deletion (CTCT) affected not only mRNA decay and transcription expression level, but also the translation expression level of its downstream gene.

Seed dormancy is a complex quantitative trait. In wheat, ABA signaling is the main factor for seed dormancy [46, 47]. *TaAFP* is a negative regulator in seed dormancy in wheat. In the present study, an STS marker AFPB associated with seed dormancy in Chinese wheat cultivars with different GI values was developed. In this set of germplasms, there are significant differences in GI between the *TaAFP-B1a* and *TaAFP-B1b* genotypes (*P* = 0.0002). Several STS markers like *Vp1A3*, *Vp1B3*, *TaSdr* and *Tamyb10D* were associated with seed dormancy in wheat [21, 22, 25]. The *TaVp-1*, *TaAFP*, and *TaSdr* genes were involved in the mechanism of embryo-imposed dormancy, while *Tamyb10* was involved in the mechanism of coat-imposed dormancy. It is better to obtain highly efficient marker-assisted selection for PHS-resistant varieties by combining both the embryo-imposed and coat-imposed dormancy.

## Conclusions

In this study, two allelic variations of *TaAFP-B* were identified, including *TaAFP-B1a* and *TaAFP-B1b*. *TaAFP-B* had a 4-bp InDel in the 5’UTR, which affected the mRNA stability, mRNA transcription expression level, translation expression level of *tdTomatoER* and *GUS*, and GUS activity and was significantly associated with PHS tolerance in common wheat. Based on the 4-bp InDel, a functional marker was developed and validated.

## Materials and methods

### Plant materials

Ten wheat varieties were used for cloning of *TaAFP-A*, *TaAFP-B*, and *TaAFP-D*, including five PHS-resistant varieties (Xiaoyan6, Langzhongbaimaizi, Wanxianbaimaizi, Yangxiaomai, and Xiaobaiyuhua, with GI values of 0.137, 0.084, 0.076, 0.075, and 0.04, respectively) and five PHS-susceptible varieties (Yongchuanbaimaizi, Xinong 979, Zhoumai 16, Zhou 8425B, and Jinan 16, with GI values of 0.232, 0.327, 0.494, 0.560, and 0.616, respectively). Ninety-one Chinese wheat varieties and advanced lines with different PHS tolerances, from the China Autumn-sown Wheat Region (CAWR) representing more than 85% of wheat production areas in China, were used for association analysis of *TaAFP-B* allelic variations and PHS tolerance. The GI was determined based on the average date across two cropping seasons at two locations in Beijing and Anyang (Henan) in 2005–2006 and 2006–2007 (Additional file 4: Table S1). Grains of Zhou 8425B and Wanxianbaimaizi planted in Hohhot in Inner Mongolia after seeds vernalization in 2016, and collected the spikes at 10, 20, 30, and 40 DAP, and at maturity were frozen in liquid nitrogen, and stored at − 80 °C for analysis of mRNA transcription. Gene gun transformation was conducted during the period when there was one leaf and one terminal bud of wheat.

### Primer design

Nine gene-specific primers, TaAFP-AF1/R1, TaAFP-AF2/R2, TaAFP-AF3/R3, TaAFP-BF1/R1, TaAFP-BF2/R2, TaAFP-BF3/R3, TaAFP-DF1/R1, TaAFP-DF2/R2, and TaAFP-DF3/R3, were used to clone *TaAFP-A*, *TaAFP-B*, and *TaAFP-D* genes (Table 1). Another primer AFPBF/R was a specific marker used for determining the allelic variants of *TaAFP-B* detected by 10% denaturing polyacrylamide gels. The primer Q-TaAFP-BF/R, Q-TaAFP-AF/R, Q-TaAFP-DF/R, and Q-ABI5F/R were designed to perform analysis of mRNA expression levels of *TaAFP-A*, *TaAFP-B*, *TaAFP-D*, and *TaABI5*. The wheat *ACTIN* gene was used as an internal control and included in each reaction in order to normalize the expression levels of *TaAFP-A*, *TaAFP-D*, and *TaABI5* genes in Zhou 8425B and Wanxianbaimaizi, and the expected PCR product was 410-bp in length (Table 1).

### DNA and RNA extraction

Genomic DNA was extracted from 3 g of seedlings grown in the dark at 25 °C for 7 days by the CTAB method [48]. Total RNA was extracted from five whole grains at different developmental stages with the TransZol Plant Kit (TransGenBiotech).

### PCR amplification and RT-qPCR analysis

PCR reactions for gene cloning and molecular marker tests were performed in an MJ Research PTC-200 thermal cycler in a total volume of 15 μl, including 1.5 μl of 10 × PCR buffer, 1.2 μl of 2.5 μM dNTP each, 4 pmol of each primer, 0.75 U of La*Taq* polymerase (TaKaRa), and 500 ng of template DNA, then up to 15 μl with ddH_2_O. PCR amplification were 94 °C for 5 min, followed by 35 cycles of 94 °C for 45 s, 57 °C–65 °C for 45 s, and 72 °C for 45 s, with a final extension of 72 °C for 10 min. Amplified PCR fragments were separated on 1.5% agarose gel with the nucleic acid dye Gelview (TaKaRa).

The cDNA was synthesized from 5 μg of total RNA using M-MLV reverse transcriptase (TaKaRa) with random hexamer primer Oligo (dT)_19_ according to the manufacturer’s instructions. RT-qPCR reactions were performed in a LightCycler®480 Real Time PCR System following the introduction book of SYRB® Premix Ex Taq™ II (TLiRnaseH Plus). PCR cycling was performed at 94 °C for 5 min, followed by 40 cycles of 94 °C for 30 s, 55 °C − 66 °C for 30 s and 72 °C for 30 s, with a final extension at 72 °C for 10 min. Then, the expression level of the *TaAFP-B* gene was estimated based on the size of the 102-bp fragment amplified with Q-TaAFP-BF/R primers designed in the exon region. The gene transcriptional expression analysis of *TaAFP* and *TaABI5* were also estimated also with RT-qPCR; which primers showed in Table 1.

### mRNA half-life assay

The *TaAFP-B* mRNA half-life (t_1/2_) was determined in flag leaves at the heading stage, dry mature seeds immersed in water for 24 h and seeds at 20 DAP of cultivars Zhou 8425B (*TaAFP-B1a*) and Wanxianbaimaizi (*TaAFP-B1b*), treated with 200 ng/mL Cordycepin (Sigma-Genosys, USA) for 0 min and 60 mins, respectively. The mRNA half-life (t_1/2_) was calculated based on the formula: T(1/2) = 0.693/kd [49], where the denominator kd is a gradient constant determined by the value of Ct in the real-time RT-PCR reaction.

### DNA sequencing

The PCR products were sequenced from both strands by the Beijing Genomics Institute (http://www.genomics.cn). Sequence analysis and characterization were performed using the software DNAMAN (http://www.lynnon.com).

### Statistical analysis

Analysis of variance was conducted by PROC MIXED in the Statistical Analysis System (SAS Institute, 8.0) with genotype clusters indicated by two types of fragments amplified with the STS marker AFPB, as a categorical variable to derive mean GI value from each cluster and to test significance levels. The genotype clusters were treated as fixed effects, while genotypes nested in clusters and years were treated as random effects. Pearson’s linear correlation coefficients for GI between years were obtained by SAS PROC CORR.

### Plasmid constructions

**Fig. 7:**
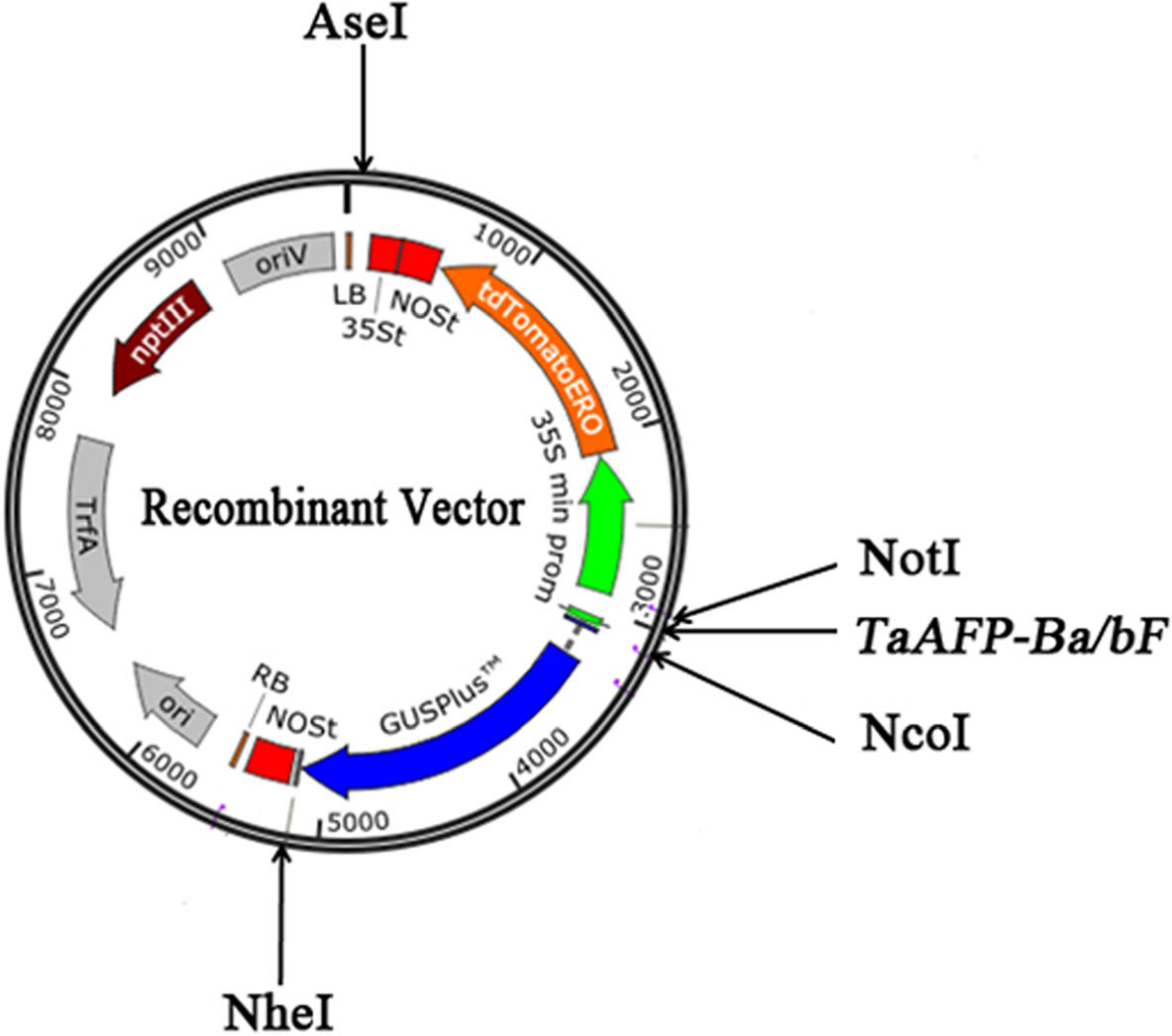
Illustration of recombinant plasmid construction in the transient experssion experiment

The plant expression vector of pITV1#1534 was supplied by YP. Xing from Inner Mongolia Agricultural University. Then, the two different fragments (272 bp and 276 bp, the sequences were part of the 5’UTR of *TaAFP-B1a* and *TaAFP-B1b*, respectively*,* with an added sequence of a mini promoter in a 5′ orientation), which differ in the 4 bp deletion (CTCT), were synthesized by General Biosystems (Anhui) Co. Ltd. The confirmed sequences were cloned into *Not*I and *Noc*I sites of the binary vector pITV1#1534. Finally, two recombinant expression vectors, pITV1-*TaAFP-BaF* and pITV1-*TaAFP-BbF*, were constructed successfully (Fig. 7).

### Plant transformation and observation

When the leaves of wheat grew to the period of one leaf and one terminal bud, the first leaf was cut off about 4 cm from the tip of the leaf, and soaked in 70% ethanol for 3 min. Then, the leaves were washed with distilled water 3 times, the leaf surface was cleaned, and the leaves were attached to a piece of glass. Finally, plasmid DNA (1 μg/μl) were transferred into leaves by biolistic bombardment (PDS-1000/He system of BioRad) transformation. He pressure of the rupture disc was 1100 psi and vacuum degree was 28 inHg. The bombardment distance was 9 cm [50, 51]. Every experiment had three biological replicates.

The tdTomatoER expression was viewed in whole leaves mounted on glass slides using the Olympus BX-60 of Confocal Laser Scanning Microscope (excitation light 540 nm, emitted light 580 nm). Images were processed with Adobe Photoshop (Mountain View, CA). Then, for observation of GUS expression, the samples were incubated overnight in a solution of 1 mM X-Gluc in 50 mM phosphate buffer (pH 7.0) at 37 °C. After that, tissues were cleared of chlorophyll in 70% ethanol and photographs of whole-mounted tissues were taken using an optical microscope. Finally, all transgenic plants for each construct were analyzed.

### Quantitative GUS assay

All transient expression wheat leaves separately converted to pITV1-*TaAFP-BaF*, pITV1-*TaAFP-BbF*, and pITV1#1534 as a control by biolistic bombardment were frozen in liquid nitrogen, and stored at − 80 °C prior to the quantitative analysis of GUS activity of the promoter of *TaAFP-BaF* and *TaAFP-BbF*, which was expressed as nanomoles 4-MU (4-methylumbelliferone) per minute per microgram protein [52]. For each construct, at least six independent transient expression wheat leaves were analyzed, and three replicates were performed.

## Additional files

Additional file 1: **Figure S1.** Sequence comparison of two new *TaAFP-A* alleles of *TaAFP-A1a* and *TaAFP-A1b* detected in Chinese germplasm with *TaAFP-A* (AB360911).SNPs are in bold letters. (DOCX 19 kb)

Additional file 2: **Figure S2.** Sequence comparison of three new *TaAFP-B* alleles of *TaAFP-B1a*, *TaAFP-B1c* and *TaAFP-B1b* detected in Chinese germplasm with *TaAFP-B* (AB360912). Insertions are underlined, deletions are shadowed, and SNPS are in bold letters. (DOCX 17 kb)

Additional file 3: **Figure S3.** Sequence comparison of two new *TaAFP-D* alleles of *TaAFP-D1a* and *TaAFP-D1b* detected in Chinese germplasm with *TaAFP-D* (AB360913). SNPS are in bold letters. (DOCX 19 kb)

Additional file 4: **Table S1.** Polymorphism of the AFPB marker in 91 white-grained cultivars with different levelsof seed dormancy. (DOCX 20 kb)

## Data Availability

The data sets supporting the results of this article are included within the article and its additional files. Sequence data used in this manuscript can be found in datebase of NCBI (https://www.ncbi.nlm.nih.gov/) under the following accession numbers: *TaAFP-A*(AB360911), *TaAFP-B*(AB360912), *TaAFP-D*(AB360913).

## Abbreviations

ABA: Abscisic acid
GA: Gibberellins
LEA: Late embryo genesis abundant
PHS: Pre-harvest sprouting
*Vp-1*: *Viviparous-1*
