# Supplementary material for: A 4-bp deletion in the 5’UTR of TaAFP-B is associated with seed dormancy in common wheat (Triticum aestivum L.)
Source: BMC Plant Biol. 2019 Aug 9;19:349. doi: 10.1186/s12870-019-1950-4 (PMC6688260; doi:10.1186/s12870-019-1950-4)
Supplement: Supplementary file 1 — Figure S1. Sequence comparison of two new TaAFP-A alleles of TaAFP-A1a and TaAFP-A1b detected in Chinese germplasm with TaAFP-A (AB360911).SNPs are in bold letters. (DOCX 19 kb) [file 12870_2019_1950_MOESM1_ESM.docx]

AB360911 GGGATCTGATTGCCGCCGATTCTACTGGCTCTGCTTGCCCGGCGCTCGTCGATTGTTTGT 1320

*TaAFP-A1a* ............................................GATTCTACTGGCTCTGCTTGCCCGGCGCTCGTCG**G**TTGTTTGT 43

*TaAFP-A1b* ............................................GATTCTACTGGCTCTGCTTGCCCGGCGCTCGTCGATTGTTTGT 43

*TaAFP-A*  TCCTCCCTGGGCGCTGTGATGTTCTTCCCTCTGGGTTTGGAGACCGAGAATTGCCCCGCG 1380

*TaAFP-A1a* TCCTCCCTGGGCGCTGTGATGTTCTTCCCTCTGGGTTTGGAGACCGAGAATTGCCCCGCG 103

*TaAFP-A1b* TCCTCCCTGGGCGCTGTGATGTTCTTCCCTCTGGGTTTGGAGACCGAGAATTGCCCCGCG 103

AB360911 CGGGACGGATTCGCGGCAATGGCGAGGCGTTCCTGAGAATTTGGCCGTCCTTGGAGCTTC 1440

*TaAFP-A1a* CGGGACGGATTCGCGGCAATGGCGAGGCGTTCCTGAGAATTTGGCCGTCCTTGGAGCTTC 163

*TaAFP-A1b* CGGGACGGATTCGCGGCAATGGCGAGGCGTTCCTGAGAATTTGGCCGTCCTTGGAGCTTC 163

AB360911 GGGGGAGGAGGGATTGGCGTGTGCTTCGCGGCGGGGAATTGCTTGCTCTCTCTCTCTGCC 1500

*TaAFP-A1a* GGGGGAGGAGGGATTGGCGTGTGCTTCGCGGCGGGGAATTGCTTGCTCTCTCTCTCTGCC 223

*TaAFP-A1b* GGGGGAGGAGGGATTGGCGTGTGCTTCGCGGCGGGGAATTGCTTGCTCTCTCTCTCTGCC 223

AB360911 GGACGGACATGGCGTCGAGGGACTTCTTGGGCAGGTTCGGCGGGGAGAAGGGGGCGGCGT 1560

*TaAFP-A1a* GGACGGACATGGCGTCGAGGGACTTCTTGGGCAGGTTCGGCGGGGAGAAGGGGGCGGCGT 283

*TaAFP-A1b* GGACGGACATGGCGTCGAGGGACTTCTTGGGCAGGTTCGGCGGGGAGAAGGGGGCGGCGT 283

AB360911 CGGACAAGGCGGGGGGCGGCGCCGGCGAGGCGGACGAGGTGGCCGAGCTCAGCCTCGGCC 1620

*TaAFP-A1a* CGGACAAGGCGGGGGGCGGCGCCGGCGAGGCGGACGAGGTGG**T**CGAGCTCAGCCTCGGCC 343

*TaAFP-A1b* CGGACAAGGCGGGGGGCGGCGCCGGCGAGGCGGACGAGGTGG**T**CGAGCTCAGCCTCGGCC 343

AB360911 TGTCCCTGGGCGGCTGCTTCGGCGCCAACTCCGGCCGGGACGCCAAGAAGCCGCGGCTGG 1680

*TaAFP-A1a* TGTCCCTGGGCGGCTGCTTCGGCGCCAACTCCGGCCGGGACGCCAAGAAGCCGCGGCTGG 403

*TaAFP-A1b* TGTCCCTGGGCGGCTGCTTCGGCGCCAACTCCGGCCGGGACGCCAAGAAGCCGCGGCTGG 403

AB360911 TGCGCTCCTCCTCCCTCGCCGCCATGTGCTCGCTCCCGGGCACCAGCGACGACCTCGCCG 1740

*TaAFP-A1a* TGCGCTCCTCCTCCCTCGCCGCCATGTGCTCGCTCCCGGGCACCAGCGACGACCTCGCCG 463

*TaAFP-A1b* TGCGCTCCTCCTCCCTCGCCGCCATGTGCTCGCTCCCGGGCACCAGCGACGACCTCGCCG 463

AB360911 CCGCGACGCCCCCGCCGGCGCCGCTGATGCGCACCAGCTCGCTCCCCACCGAGACCGAGG 1800

*TaAFP-A1a* CCGCGACGCCCCCGCCGGCGCCGCTGATGCGCACCAGCTCGCTCCCCACCGAGACCGAGG 523

*TaAFP-A1b* CCGCGACGCCCCCGCCGGCGCCGCTGATGCGCACCAGCTCGCTCCCCACCGAGACCGAGG 523

AB360911 AGGAGCGGTGGCGCCGGCGCGAGATGCAGAGCCTCAAGCGCCTCCAGGCCAAGCGCAAGC 1860

*TaAFP-A1a* AGGAGCGGTGGCGCCGGCGCGAGATGCAGAGCCTCAAGCGCCTCCAGGCCAAGCGCAAGC 583

*TaAFP-A1b* AGGAGCGGTGGCGCCGGCGCGAGATGCAGAGCCTCAAGCGCCTCCAGGCCAAGCGCAAGC 583

AB360911 GCCTCGAGCGCCGCACCTCCATGAACTCCGGCAAGTCCGGCGGCAGCAGCAGCCGGGACG 1920

*TaAFP-A1a* GCCTCGAGCGCCGCACCTCCATGAACTCCGGCAAGTCCGGCGGCAGCAGCAGCCGGGACG 643

*TaAFP-A1b* GCCTCGAGCGCCGCACCTCCATGAACTCCGGCAAGTCCGGCGGCAGCAGCAGCCGGGACG 643

AB360911 ACGCCCAGGAGCCGCTCTACCCCAGCGCGTTCCAGCTCCGCCGCTCCGTCGTCGACCAGG 1980

*TaAFP-A1a* ACGCCCAGGAGCCGCTCTACCCCAGCGCGTTCCAGCTCCGCCGCTCCGTCGTCGACCAGG 703

*TaAFP-A1b* ACGCCCAGGAGCCGCTCTACCCCAGCGCGTTCCAGCTCCGCCGCTCCGTCGTCGACCAGG 703

AB360911 GGAACCCCTCCTCAAGCATGCCCGAGCAAGGTATGCACATGCTTTCACCAACTATTCGCT 2040

*TaAFP-A1a* GGAACCCCTCCTCAAGCATGCCCGAGCAAGGTATGCACATGCTTTCACCAACTATTC**T**CT 763

*TaAFP-A1b* GGAACCCCTCCTCAAGCATGCCCGAGCAAGGTATGCACATGCTTTCACCAACTATTCGCT 763

AB360911 ACAATCAGCTTGCAATTCCCACTGTTCTATTGCGCTTCCTTGCTCGAATTATTCATCCAG 2100

*TaAFP-A1a* ACAATCAGCTTGCAATTCCCACTGTTCTATTGCGCTTCCTTGCTCGAATTATTCATCCAG 823

*TaAFP-A1b* ACAATCAGCTTGCAATTCCCACTGTTCTATTGCGCTTCCTTGCTCGAATTATTCATCCAG 823

AB360911 CTGCCTGTTAAGCTCTCGGCGATCCATGCAGGTCGGTGTCATGCCGAGCTCACCGTAGTT 2160

*TaAFP-A1a* CTGCCTGTTAAGCTCTCGGCGATCCATGCAGGTCGGTGTCATGCCGAGCTCACCGTAGTT 883

*TaAFP-A1b* CTGCCTGTTAAGCTCTCGGCGATCCATGCAGGTCGGTGTCATGCCGAGCTCACCGTAGTT 883

AB360911 CTTTCGGTAGGAGATGCATGGGCAGAGGGGGGATCTAAAAAGTCGAGTGTTCTTTGCCAT 2220

*TaAFP-A1a* CTTTCGGTAGGAGATGCATGGGCAGAGGGGGGATCTAAAAAGTCGAGTGTTCTTTGCCAT 943

*TaAFP-A1b* CTTTCGGTAGGAGATGCATGGGCAGAGGGGGGATCTAAAAAGTCGAGTGTTCTTTGCCAT 943

AB360911 GGATCTTGCTTTGGTATTGTGATTTACCGGGATCGATTCGTTAGAACGCTAGCTCAGCCG 2280

*TaAFP-A1a* GGATC**A**TGCTTTGGTATTGTGATTTACCGGGATCGATTCGTTAGAACGCTAGCTCAGCCG 1003

*TaAFP-A1b* GGATC**A**TGCTTTGGTATTGTGATTTACCGGGATCGATTCGTTAGAACGCTAG**A**TCAGCCG 1003

AB360911 ATGCTTTTCTTTTTCGCCAAATTCGTTAGAAATGGGGAATTCTTTTCTGCACAACCTGAT 2340

*TaAFP-A1a* ATGCTTTTCTTTTTCGCCAAATTCGTTAGAAATGGGGAATTCTTTTCTGCACAACCTGAT 1063

*TaAFP-A1b* ATGCTTTTCTTTTTCGCCAAATTCGTTAGAAATGGGGAATTCTTTTCTGCACAACCTGAT 1063

AB360911 GATGCTTCCACGTACGCATGGGATTTGTTGTGTTCTTTTCGGGGCGTTTGTTTTGGGTGA 2400

*TaAFP-A1a* GATGCTTCCACGTACGCATGGGATTTGTTGTGTTCTTTTCGGGGCGTTTGTTTTGGGTGA 1123

*TaAFP-A1b* GATGCTTCCACGTACGCATGGGATTTGTTGTGTTCTTTTCGGGGCGTTTGTTTTGGGTGA 1123

AB360911 TGTCATTTCTGGGATTATTTCGAGCCGTGCTGTTGCTCCTAGGGTCTGGAGAGATGCCTT 2460

*TaAFP-A1a* TGTCATTTCTGGGATTATTTCGAGCCGTGCTGTTGCTCCTAGGGTCTGGAGAGATGCCTT 1183

*TaAFP-A1b* TGTCATTTCTGGGATTATTTCGAGCCGTGCTGTTGCTCCTAGGGTCTGGAGAGATGCCTT 1183

AB360911 CTACGGCATGTTTCTAAGCAGCTTCTAAGCTTTTAGTCACTACTAGTCAGTAGTTACGTA 2520

*TaAFP-A1a* CTACGGCATGTTTCTAAGCAGCTTCTAA**A**CTTTTAGTCACTACTAGTCAGTAGTTACGTA 1243

*TaAFP-A1b* CTACGGCATGTTTCTAAGCAGCTT**A**TAAGCTTTTAGTCACTACTAGTCAGTAGTTACGTA 1243

AB360911 GCGTCAGAGTCTGATTATTGAGGCTAATCACTATCCGAGCAGCTGCTAAACATATATGAT 2580

*TaAFP-A1a* GCGTCAGAGTCTGATTATTGAGGCTAATCACTATCCGAGCAGCTGCTAAACATATATGAT 1303

*TaAFP-A1b* GCGTCAGAGTCTGATTATTGAGGCTAATCACTATCCGAGCAGCTGCTAAACATATATGAT 1303

AB360911 TTGAATTGTCTACCTGAAAAACTGGAAAGCACTGACGATTAGTAAACGGAAAAGAGCACA 2640

*TaAFP-A1a* TTGAATTGTCTACCTGAAAAACTGGAAAGCACTGACGATTAGTAAACGGAAAAGAGCACA 1363

*TaAFP-A1b* TTGAATTGTCTACCTGAAAAACTGGAAAGCACTGACGATTAGTAAACGGAAAAGAGCACA 1363

AB360911 TTGGGATATGAATCTTTATTGACATGTTGGAATATGAAAATGTGACATCTCCTCCTCCTC 2700

*TaAFP-A1a* TTGGGATATGAATCTTTATTGACATGTTGGAATATGAAAATGTGACATCTCCTCCTCCTC 1423

*TaAFP-A1b* TTGGGATATGAATCTTTATTGACATGTTGGAATATGAAAATGTGACATCTCCTCCTCCTC 1423

AB360911 TGCATTGGTGCAGGTAGCGGTGATGGCGCTGAGGTGAAGAGCACATCGAGCATGGAGATA 2760

*TaAFP-A1a* TGCATTGGTGCAGGTAGCGGTGATGGCGCTGAGGTGAAGAGCACATCGAGCATGGAGATA 1483

*TaAFP-A1b* TGCATTGGTGCAGGTAGCGGTGATGGCGCTGAGGTGAAGAGCACATCGAGCATGGAGATA 1483

AB360911 TCTTCCGATAATAATAACAATGCCAGCAACCAGAACAAATCCCTCCCGCCGCCGGCACCA 2820

*TaAFP-A1a* TCTTCCGATAATAATAACAATGCCAGCAACCAGAACAAATCCCTCCCGCCGCCGGCACCA 1543

*TaAFP-A1b* TCTTCCGATAATAATAACAATGCCAGCAACCAGAACAAATCCCTCCCGCCGCCGGCACCA 1543

AB360911 TCTCCGGCGGCCGGGAAGCTGCCGAACGGCATCGTCAAGGAGCAACCGCCGTTGCGGACC 2880

*TaAFP-A1a* TCTCCGGCGGCCGGGAAGCTGCCGAACGGCATCGTCAAGGAGCAACCGCCGTTGCGGACC 1603

*TaAFP-A1b* TCTCCGGCGGCCGGGAAGCTGCCGAACGGCATCGTCAAGGAGCAACCGCCGTTGCGGACC 1603

AB360911 CTCCGGTCGCTGACGATGCGCACGACGAGCACCGGCGACCTGCGGAAGAGCATGATGGAG 2940

*TaAFP-A1a* CTCCGGTCGCTGACGATGCGCACGACGAGCACCGGCGACCTGCGGAAGAGCATGATGGAG 1663

*TaAFP-A1b* CTCCGGTCGCTGACGATGCGCACGACGAGCACCGGCGACCTGCGGAAGAGCATGATGGAG 1663

AB360911 GACATGCCGATGGTCTCGTCCAAGGTGGACGGCCCCAACGGCAAGAAGATCGACGGCTTC 3000

*TaAFP-A1a* GACATGCCGATGGTCTCGTCCAAGGTGGACGGCCCCAACGGCAAGAAGATCGACGGCTTC 1723

*TaAFP-A1b* GACATGCCGATGGTCTCGTCCAAGGTGGACGGCCCCAACGGCAAGAAGATCGACGGCTTC 1723

AB360911 CTGTACAAGTACAGGAAAGGGGAGGAGGTGAGGATAGTGTGCGTTTGCCACGGCAACTTC 3060

*TaAFP-A1a* CTGTACAAGTACAGGAAAGGGGAGGAGGTGA**A**GATAGTGTGCGTTTGCCACGGCAACTTC 1783

*TaAFP-A1b* CTGTACAAGTACAGGAAAGGGGAGGAGGTGAGGATAGTGTGCGTTTGCCACGGCAACTTC 1783

Fig S1. Sequence comparison of two new *TaAFP-A* alleles of *TaAFP-A1a* and *TaAFP-A1b* detected in Chinese germplasm with *TaAFP-A*(AB360911).SNPs are in bold letters
