# Supplementary material for: A 4-bp deletion in the 5’UTR of TaAFP-B is associated with seed dormancy in common wheat (Triticum aestivum L.)
Source: BMC Plant Biol. 2019 Aug 9;19:349. doi: 10.1186/s12870-019-1950-4 (PMC6688260; doi:10.1186/s12870-019-1950-4)
Supplement: Supplementary file 2 — Figure S2. Sequence comparison of three new TaAFP-B alleles of TaAFP-B1a, TaAFP-B1c and TaAFP-B1b detected in Chinese germplasm with TaAFP-B (AB360912). Insertions are underlined, deletions are shadowed, and SNPS are in bold letters. (DOCX 17 kb) [file 12870_2019_1950_MOESM2_ESM.docx]

AB360912 CTCGCTCGAGGATCCCACACGCCGCGCGCAGGGATCTGATTGCCGCCGATTCTGCTGGCT 1860

*TaAFP-B1a* ................................................................................................................................GATTCTGCTGGCT 13

*TaAFP-B1c* .................................................................................................................................GATTCTGCTGGCT 13

*TaAFP-B1b* ................................................................................................................................GATTCTGCTGGCT 13

*AB360912* CTGCTTCCCCGGCGCTCGTCGACTGTTCGTTCCTCCCTGGGCGCTGTGATGTTCTTCCCT 1920

*TaAFP-B1a* CTGCTTCCCCGGCGCTCGTCGA..TGTTCGTTCCTCCCTGGGCGCTGTGATGTTCTTCCCT 73

*TaAFP-B1c* CTGCTTCCCCGGCGCTCGTCGA..**C**TGTTCGTTCCTCCCTGGGCGCTGTGATGTTCTTCCCT 73

TaAFP-B1b CTGCTTCCCCGGCGCTCGTCGA..TTGTTCGTTCCTCCCTGGGCGCTGTGATGTTCTTCCCT 73

AB360912 CTGGGTTTGGAGACCGAGAATTGCCCGGCGCGGGACGGATTCGCGGCAATGGCGAGGTCT 1980

*TaAFP-B1a* CTGGGTTTG**A**AGACCGAGAATTGCCCGGCGCGGGACGGATTCGCGGCAATGGCGAGGTCT 133

*TaAFP-B1c* CTGGGTTTGGAGACCGAGAATTGCCCGGCGCGGGACGGATTCGCGGCAATGGCGAGGTCT 133

*TaAFP-B1b* CTGGGTTTGGAGACCGAGAATTGCCCGGCGCGGGACGGATTCGCGGCAATGGCGAGGTCT 133

AB360912 TCCTGAGAATTTGGCCGTCCTTGGAGGTTGGGGGGAGGAGGGATTGGCGTCTGCTTC..CG 2039

*TaAFP-B1a* TCCTGAGAATTTGGCCGTCCTTGGAGGTTGGGGGGAGGAGGGATTGGCGTCTGCTTCGCG 193

*TaAFP-B1c* TCCTGAGAATTTGGCCGTCCTTGGAGGTTGGGGGGAGGAGGGATTGGCGTCTGCTTCGCG 193

*TaAFP-B1b* TCCTGAGAATTTGGCCGTCCTTGGAGGTTGGGGGGAGGAGGGATTGGCGTCTGCTTCGCG 193

AB360912 GCGGGGAATTGCTTG....CTTGCTCTCTCTCTCTGCCGGACGGACATGGCGTCGAGGGAC 2096

*TaAFP-B1a* GCGGGGAATTGCTTGCTCTCTCTCTCTCTCTCTGCCGGACGGACATGGCGTCGAGGGAC 252

*TaAFP-B1c* GCGGGGAATTGCTTGCTCTCTCTCTCTCTCTCTGCCGGACGGACATGGCGTCGAGGGAC 252

*TaAFP-B1b* GCGGGGAATTGCTTG..........CTCTCTCTCTCTCTGCCGGACGGACATGGCGTCGAGGGAC 248

AB360912 TTCTTGGGCAGGTTCGGCGGCGAGAAGGGCTCGTCGTCGGACAAGGCGGGGGGCGGCGCC 2156

*TaAFP-B1a* TTCTTGGGCAGGTTCGGCGGCGAGAAGGGCTCGTCGTCGGACAAGGCGGGGGGCGGCGCC 312

*TaAFP-B1c* TTCTTGGGCAGGTTCGGCGGCGAGAAGGGCTCGTCGTCGGACAAGGCGGGGGGCGGCGCC 312

*TaAFP-B1b* TTCTTGGGCAGGTTCGGCGGCGAGAAGGGCTCGTCGTCGGACAAGGCGGGGGGCGGCGCC 308

AB360912 GGCGAGCCCGACGAGGTGGTCGAGCTCAGCCTGGGCCTGTCCCTGGGCGGCTGCTTCGGC 2216

*TaAFP-B1a* GGCGAGCCCGACGAGGTGGTCGAGCTCAGCCTGGGCCTGTCCCTGGGCGGCTGCTTCGGC 372

*TaAFP-B1c* GGCGAGCCCGACGAGGTGGTCGAGCTCAGCCTGGGCCTGTCCCTGGGCGGCTGCTTCGGC 372

*TaAFP-B1b* GGCGAGCCCGACGAGGTGGTCGAGCTCAGCCTGGGCCTGTCCCTGGGCGGCTGCTTCGGC 368

AB360912 GCCAACTCCGGCCGGGACGCCAAGAAGCCGCGGCTGGTGCGCTCCTCCTCCCTCGCCGCC 2276

*TaAFP-B1a* GCCAACTCCGGCCGGGACGCCAAGAAGCCGCGGCTGGTGCGCTCCTCCTCCCTCGCCGCC 432

*TaAFP-B1c* GCCAACTCCGGCCGGGACGCCAAGAAGCCGCGGCTGGTGCGCTCCTCCTCCCTCGCCGCC 432

*TaAFP-B1b* GCCAACTCCGGCCGGGACGCCAAGAAGCCGCGGCTGGTGCGCTCCTCCTCCCTCGCCGCC 428

AB360912 ATGTACTCGCTCCCGGGCACCAGCGACGACCTCGCCGCCGCCACGCCCCCGCCGGCGCCG 2336

*TaAFP-B1a* ATGT**G**CTCGCTCCCGGGCACCAGCGACGACCTCGCCGCCGCCACGCCCCCGCCGGCGCCG 492

*TaAFP-B1c* ATGT**G**CTCGCTCCCGGGCACCAGCGACGACCTCGCCGCCGCCACGCCCCCGCCGGCGCCG 492

*TaAFP-B1b* ATGTACTCGCTCCCGGGCACCAGCGACGACCTCGCCGCCGCCACGCCCCCGCCGGCGCCG 488

AB360912 CTGATGCGCACCAGCTCGCTCCCCACCGAGACGGAGGAGGAGCGGTGGCGCCGCCGCGAG 2396

*TaAFP-B1a* CTGATGCGCACCAGCTCGCTCCCCACCGAGACGGAGGAGGAGCGGTGGCGCCGCCGCGAG 552

*TaAFP-B1c* CTGATGCGCACCAGCTCGCTCCCCACCGAGACGGAGGAGGAGCGGTGGCGCCGCCGCGAG 552

*TaAFP-B1b* CTGATGCGCACCAGCTCGCTCCCCACCGAGACGGAGGAGGAGCGGTGGCGCCGCCGCGAG 548

AB360912 ATGCAGAGCCTCAAGCGCCTCCAGGCCAAGCGCAAGCGCCTCGAGCGCCGCACCTCCATG 2456

*TaAFP-B1a* ATGCAGAGCCTCAAGCGCCTCCAGGCCAAGCGCAAGCGCCTCGAGCGCCGCACCTCCATG 612

*TaAFP-B1c* ATGCAGAGCCTCAAGCGCCTCCAGGCCAAGCGCAAGCGCCTCGAGCGCCGCACCTCCATG 612

*TaAFP-B1b* ATGCAGAGCCTCAAGCGCCTCCAGGCCAAGCGCAAGCGCCTCGAGCGCCGCACCTCCATG 608

AB360912 AACTCCGGCAAGTCCGGCGGCAGCAGCAGCCGGGACGACGCCCAGGAGCCGCTCTACCCC 2516

*TaAFP-B1a* AACTCCGGCAAGTCCGGCGGCAGCAGCAGCCGGGACGACGCCCAGGAGCCGCTCTACCCC 672

*TaAFP-B1c* AACTCCGGCAAGTCCGGCGGCAGCAGCAGCCGGGACGACGCCCAGGAGCCGCTCTACCCC 672

*TaAFP-B1b* AACTCCGGCAAGTCCGGCGGCAGCAGCAGCCGGGACGACGCCCAGGAGCCGCTCTACCCC 668

AB360912 AGCGCGTTCCAGCTCCGCCGCTCCGTCGTCGACCAGGGGAACACCTCCTCAAGCATGCCG 2576

*TaAFP-B1a* AGCGCGTTCCAGCTCCGCCGCTCCGTCGTCGACCAGGGGAACACCTCCTCAAGCATGCCG 732

*TaAFP-B1c* AGCGCGTTCCAGCTCCGCCGCTCCGTCGTCGACCAGGGGAACACCTCCTCAAGCATGCCG 732

*TaAFP-B1b* AGCGCGTTCCAGCTCCGCCGCTCCGTCGTCGACCAGGGGAACACCTCCTCAAGCATGCCG 728

AB360912 GAGCAAGGTATACACATGCTTTCATCAGCTTCCCTACCACTCGAACTGTTTGCTACAATA 2636

*TaAFP-B1a* GAGCAAGGTATACACATGCTTTCATCAGCTTCCCTACCACTCGAACTGTTTGCTACAATA 792

*TaAFP-B1c* GAGCAAGGTATACACATGCTTTCATCAGCTTCCCTACCACTCGAACTGTTTGCTACAATA 792

*TaAFP-B1b* GAGCAAGGTATACACATGCTTTCATCAGCTTCCCTACCACTCGAACTGTTTGCTACAATA 788

AB360912 AGCTTGCAATTCCCATTGTTCTATTGCGTTCCCTTGCTTGAATTATTCATTCAGCTGCCT 2696

*TaAFP-B1a* AGCTTGCAATTCCCA**C**TGTTCTATTGCGTTCCCTTGCTTGAATTATTCATTCAGCTGCCT 852

*TaAFP-B1c* AGCTTGCAATTCCCA**C**TGTTCTATTGCGTTCCCTTGCTTGAATTATTCATTCAGCTGCCT 852

*TaAFP-B1b* AGCTTGCAATTCCCA**C**TGTTCTATTGCGTTCCCTTGCTTGAATTATTCATTCAGCTGCCT 848

AB360912 GTTCTGGTTGCCAAGCTCTCGGCGATCCATGCAGGTCGGTGTCATGCCGAGCTTACCGTG 2756

*TaAFP-B1a* GTTCTGGTTGCCAAGCTCTCGGCGATCCATGCAGGTCGGTGTCATGCCGAGCTTACCGTG 912

*TaAFP-B1c* GTTCTGGTTGCCAAGCTCTCGGCGATCCATGCAGGTCGGTGTCATGCCGAGCTTACCGTG 912

*TaAFP-B1b* GTTCTGGTTGCCAAGCTCTCGGCGATCCATGCAGGTCGGTGTCATGCCGAGCTTACCGTG 908

AB360912 GTTCTTTTGGTAGGAGATGCATGGGCAGAGGGGGGATCTAGCAAGTCGAGTGTTCTTTGC 2816

*TaAFP-B1a* GTTCTTTTGGTAGGAGATGCATGGGCAGAGGGGGGATCTAGCAAGTCGAGTGTTCTTTGC 972

*TaAFP-B1c* GTTCTTTTGGTAGGAGATGCATGGGCAGAGGGGGGATCTAGCAAGTCGAGTGTTCTTTGC 972

*TaAFP-B1b* GTTCTTTTGGTAGGAGATGCATGGGCAGAGGGGGGATCTAGCAAGTCGAGTGTTCTTTGC 968

AB360912 CATGGATCTTGCTTTGGTCTTGTGATTTACTGGGATCGATTCGTTAGAACGCTAGCTGAG 2876

*TaAFP-B1a* CATGGATCTTGCTTTGGTCTTGTGATTTACTGGGATCGATTCGTTAGAACGCTAGCTGAG 1032

*TaAFP-B1c* CATGGATCTTGCTTTGGTCTTGTGATTTACTGGGATCGATTCGTTAGAACGCTAGCTGAG 1032

*TaAFP-B1b* CATGGATCTTGCTTTGGTCTTGTGATTTACTGGGATCGATTCGTTAGAACGCTAGCTGAG 1028

AB360912 CCGATGCTTTTCTTTTTCGCCAAATTCATTAGAAATGGGGAATTCTTTTCTGCACAACCT 2936

*TaAFP-B1a* CCGATGCTTTTCTTTTTCGCCAAATTCATTAGAAATGGGGAATTCTTTTCTGCACAACCT 1092

*TaAFP-B1c* CCGATGCTTTTCTTTTTCGCCAAATTCATTAGAAATGGGGAATTCTTTTCTGCACAACCT 1092

*TaAFP-B1b* CCGATGCTTTTCTTTTTCGCCAAATTCATTAGAAATGGGGAATTCTTTTCTGCACAACCT 1088

AB360912 GATGATACTTCTACGTACGCATGGGATTTGTTGTGTTCTTTTCGGGGCGTTTGTTTTGGG 2996

*TaAFP-B1a* GATGATACTTCTACGTACGCATGGGATTTGTTGTGTTCTTTTCGGGGCGTTTGTTTTGGG 1152

*TaAFP-B1c* GATGATACTTCTACGTACGCATGGGATTTGTTGTGTTCTTTTCGGGGCGTTTGTTTTGGG 1152

*TaAFP-B1b* GATGATACTTCTACGTACGCATGGGATTTGTTGTGTTCTTTTCGGGGCGTTTGTTTTGGG 1148

AB360912 TGATGTCATTTCTGGGATTATTTCGAGCCGTGCTGTTGCTCCTAGGGTCTCAAGAGATGC 3056

*TaAFP-B1a* TGATGTCATTTCTGGGATTATTTCGAGCCGTGCTGTTGCTCCTAGGGTCTCAAGAGATGC 1212

*TaAFP-B1c* TGATGTCATTTCTGGGATTATTTCGAGCCGTGCTGTTGCTCCTAGGGTCTCAAGAGATGC 1212

*TaAFP-B1b* TGATGTCATTTCTGGGATTATTTCGAGCCGTGCTGTTGCTCCTAGGGTCTCAAGAGATGC 1208

AB360912 CTTCTACGGCATGTGTCTAAGCAGTTTCTAAGCTTTTAGTCACTACTAACAGTTACGTAG 3116

*TaAFP-B1a* CTTCTACGGCATGTGTCTAAGCAGTTTCTAAGCTTTTAGTCACTACTAACAGTTACGTAG 1272

*TaAFP-B1c* CTTCTACGGCATGTGTCTAAGCAGTTTCTAAGCTTTTAGTCACTACTAACAGTTACGTAG 1272

*TaAFP-B1b* CTTCTACGGCATGTGTCTAAGCAGTTTCTAAGCTTTTAGTCACTACTAACAGTTACGTAG 1268

AB360912 CGTCAGGGTATGATTATTCAGGCTAATCATTATCCGAGCAGCTACTACTAAATATAAGTA 3176

*TaAFP-B1a* CGTCAGGGTATGATTATTCAGGCTAATCATTATCCGAGCAGCTACTACTAAATATAAGTA 1332

*TaAFP-B1c* CGTCAGGGTATGATTATTCAGGCTAATCATTATCCGAGCAGCTACTACTAAATATAAGTA 1332

*TaAFP-B1b* CGTCAGGGTATGATTATTCAGGCTAATCATTATCCGAGCAGCTACTACTAAATATAAGTA 1328

AB360912 TATGATTTGAATTGTCTACCTGAAAACTGGAAAGCACTGACGATTAGTAAACGGAAAAGA 3236

*TaAFP-B1a* TATGATTTGAATTGTCTACCTGAAAACTGGAAAGCACTGACGATTAGTAAACGGAAAAGA 1392

*TaAFP-B1c* TATGATTTGAATTGTCTACCTGAAAACTGGAAAGCACTGACGATTAGTAAACGGAAAAGA 1392

*TaAFP-B1b* TATGATTTGAATTGTCTACCTGAAAACTGGAAAGCACTGACGATTAGTAAACGGAAAAGA 1388

AB360912 ACACATTGGGATATGAATCTTTATTGACATGTTGGAATATGAAAATGTGACATCTTCTCC 3296

*TaAFP-B1a* ACACATTGGGATATGAATCTTTATTGACATGTT**A**GAATATGAAAATGTGACATCTTCTCC 1452

*TaAFP-B1c* ACACATTGGGATATGAATCTTTATTGACATGTTGGAATATGAAAATGTGACATCTTCTCC 1452

*TaAFP-B1b* ACACATTGGGATATGAATCTTTATTGACATGTTGGAATATGAAAATGTGACATCTTCTCC 1448

AB360912 CCTGCATTGGTGCAGGTAGTGCTGATGGCGCTGAGGCGAAGAGCACATCGAGCATGGAGA 3356

*TaAFP-B1a* CCTGCATTGGTGCAGGTAG**C**GCTGATGGCGCTGAGGCGAAGAGCACATCGAGCATGGAGA 1512

*TaAFP-B1c* CCTGCATTGGTGCAGGTAGTGCTGATGGCGCTG**G**GGCGAAGAGCACATCGAGCATGGAGA 1512

*TaAFP-B1b* CCTGCATTGGTGCAGGTAGTGCTGATGGCGCTG**G**GGCGAAGAGCACATCGAGCATGGAGA 1508

AB360912 TATCTTCTGATAATAATAATAATAACAATGCCAGCAACCAGAACAAGTCCCTCCCGCCGC 3416

*TaAFP-B1a* TATCTTCTGATAATAATAATAATAACAATGCCAGCAACCAGAACAAGTCCCTCCCGCCGC 1572

*TaAFP-B1c* TATCTTCTGATAATAATAATAATAACAATGCCAGCAACCAGAACAAGTCCCTCCCGCCGC 1572

*TaAFP-B1b* TATCTTCTGATAATAATAATAATAACAATGCCAGCAACCAGAACAAGTCCCTCCCGCCGC 1568

AB360912 CGGCACCATCTCCGGCCGGGAAGCTGCCGAACGGCATCGTCAAGGAGCAACCGCCGTTGC 3476

*TaAFP-B1a* CGGCACCATCTCCGGCCGGGAAGCTGCCGAACGGCATCGTCAAGGAGCAACCGCCGTTGC 1632

*TaAFP-B1c* CGGCACCATCTCCGGCCGGGAAGCTGCCGAACGGCATCGTCAAGGAGCAACCGCCGTTGC 1632

*TaAFP-B1b* CGGCACCATCTCCGGCCGGGAAGCTGCCGAACGGCATCGTCAAGGAGCAACCGCCGTTGC 1628

AB360912 GGACCCTCCGGTCGCTGACGATGCGCACGACTAGCACCGGCGACCTGCGGAAGAGCATGA 3536

*TaAFP-B1a* GGACCCTCCGGTCGCTGACGATGCGCACGACTAGCACCGGCGACCTGCGGAAGAGCATGA 1692

*TaAFP-B1c* GGACCCTCCGGTCGCTGACGATGCGCACGACTAGCACCGGCGACCTGCGGAAGAGCATGA 1692

*TaAFP-B1b* GGACCCTCCGGTCGCTGACGATGCGCACGACTAGCACCGGCGACCTGCGGAAGAGCATGA 1688

AB360912 TGGAGGACATGCCGATGGTCTCGTCCAAGGTGGACGGCCCCAACGGCAAGAAGATCGACG 3596

*TaAFP-B1a* TGGAGGACATGCCGATGGTCTCGTCCAAGGTGGACGGCCCCAACGGCAAGAAGATCGACG 1752

*TaAFP-B1c* TGGAGGACATGCCGATGGTCTCGTCCAAGGTGGACGGCCCCAACGGCAAGAAGATCGACG 1752

*TaAFP-B1b* TGGAGGACATGCCGATGGTCTCGTCCAAGGTGGACGGCCCCAACGGCAAGAAGATCGACG 1748

AB360912 GCTTCCTCTACAAGTACAGGAAAGGGGAGGAGGTGAGGATAGTGTGCGTCTGCCATGGCA 3656

*TaAFP-B1a* GCTTCCTCTACAAGTACAGGAAAGGGGAGGAGGTGAGGATAGTGTGCGTCTGCCATGGCA 1812

*TaAFP-B1c* GCTTCCTCTACAAGTACAGGAAAGGGGAGGAGGTGAGGATAGTGTGCGTCTGCCATGGCA 1812

*TaAFP-B1b* GCTTCCTCTACAAGTACAGGAAAGGGGAGGAGGTGAGGATAGTGTGCGTCTGCCATGGCA 1808

AB360912 ACTTCCTCACGCCGGCGGAGTTCGTGAAGCACGCTGGCGGCGGCGACGTCACGAACCCGC 3716

*TaAFP-B1a* ACTTCCTCACGCCGGCGGAGTTCGTGAAGCACGCTGGCGGCGGCGACGTCACGAACCCGC 1872

*TaAFP-B1c* ACTTCCTCACGCCGGCGGAGTTCGTGAAGCACGCTGGCGGCGGCGACGTCACGAACCCGC 1872

*TaAFP-B1b* ACTTCCTCACGCCGGCGGAGTTCGTGAAGCACGCTGGCGGCGGCGACGTCACGAACCCGC 1868

AB360912 TCAGGCACATCGTCGTCAACCCCGCGCCGTCGGTCTTCTTGTAATGTCGGAATGTACCTA 3776

*TaAFP-B1a* TCAGGCACATCGTCGTCAACCCCGCGCCGTCGGTCTTCTTGTAATGTCGGAATGTACCTA 1932

*TaAFP-B1c* TCAGGCACATCGTCGTCAACCCCGCGCCGTCGGTCTTCTTGTAATGTCGGAATGTACCTA 1932

*TaAFP-B1b* TCAGGCACATCGTCGTCAACCCCGCGCCGTCGGTCTTCTTGTAATGTCGGAATGTACCTA 1928

Fig S2. Sequence comparison of three new *TaAFP-B* alleles of *TaAFP-B1a*, *TaAFP-B1c* and *TaAFP-B1b* detected in Chinese germplasm with *TaAFP-B* (AB360912).Insertions are underlined, deletions are shadowed, and SNPs are in bold letters
