# Supplementary material for: A 4-bp deletion in the 5’UTR of TaAFP-B is associated with seed dormancy in common wheat (Triticum aestivum L.)
Source: BMC Plant Biol. 2019 Aug 9;19:349. doi: 10.1186/s12870-019-1950-4 (PMC6688260; doi:10.1186/s12870-019-1950-4)
Supplement: Supplementary file 3 — Figure S3. Sequence comparison of two new TaAFP-D alleles of TaAFP-D1a and TaAFP-D1b detected in Chinese germplasm with TaAFP-D (AB360913). SNPS are in bold letters. (DOCX 19 kb) [file 12870_2019_1950_MOESM3_ESM.docx]

AB360913 GCCGCCGATTGTGCTGGCTCTGCTTCCCCGGCGCTCGTCGATTGTTCGTCCGTAGGCGCT 1080

*TaAFP-D1a* ................GATTGTGCTGGCTCTGCTTCCCCGGCGCTCGTCGATTGTTCGTCCGTAGGCGCT 54

*TaAFP-D1b* ................GATTGTGCTGGCTCTGCTTCCCCGGCGCTCGTCGATTGTTCGTCCGTAGGCGCT 54

AB360913 GTGATGTTCTTCCCTCTGGGTTTGGAGACCGAGAATTGCCCGGCGCGGGACGGATTCGCG 1140

*TaAFP-D1a* GTGATGTTCTTCCCTCTGGGTTTGGAGACCGAGAATTGCCCGGCGCGGGACGGATTCGCG 114

*TaAFP-D1b* GTGATGTTCTTCCCTCTGGGTTTGGAGACCGAGAATTGCCCGGCGCGGGACGGATTCGCG 114

AB360913 GCAATGGCGAGGTGTTCCTGAGAATTTGGCCTTCTTGGAGCTTGGGGAAGGGAAGGATTG 1200

*TaAFP-D1a* GCAATGGCGAGGTGTTCCTGAGAATTTGGCCTTCTTGGAGCTTGGGGAAGGGAAGGATTG 174

*TaAFP-D1b* GCAATGGCGAGGTGTTCCTGAGAATTTGGCCTTCTTGGAGCTTGGGGAAGGGAAGGATTG 174

AB360913 GCGTCCGCTTCGCGGCGGGGAATTGCTTGCTCTCTCTCTCGTTCTCTGTGCCGGGCATGG 1260

*TaAFP-D1a* GCGTCCGCTTCGCGGCGGGGAATTGCTTGCTCTCTCTCTCGTTCTCTGTGCCGGGCATGG 234

*TaAFP-D1b* GCGTCCGCTTCGCGGCGGGGAATTGCTTGCTCTCTCTCTCGTTCTCTGTGCCGGGCATGG 234

AB360913 CGTCGAGGGACTTCTTGGGCAGGTTCGGCGGGGAGAAGGGCGCGTCGTCGGACAAGGCGG 1320

*TaAFP-D1a* CGTCGAGGGACTTCTTGGGCAGGTTCGGCGGGGAGAAGGGCGCGTCGTCGGACAAGGCGG 294

*TaAFP-D1b* CGTCGAGGGACTTCTTGGGCAGGTTCGGCGGGGAGAAGGGCGCGTCGTCGGACAAGGCGG 294

AB360913 GGGGCGGCGCCGGCGAGCCGGACGAGGTGGTCGAGCTTAGCCTGGGCCTGTCCCTGGGCG 1380

*TaAFP-D1a* GGGGCGGCGCCGGCGAGCCGGACGAGGTGGTCGAGCTTAGCCTGGGCCTGTCCCTGGGCG 354

*TaAFP-D1b* GGGGCGGCGCCGGCGAGCCGGACGAGGTGGTCGAGCTTAGCCTGGGCCTGTCCCTGGGCG 354

AB360913 GCTGCTTCGGCGCCAACTCCGGCCGGGACGCCAAGAAGCCGCGGCTGGTGCGCTCCTCCT 1440

*TaAFP-D1a* GCTGCTTCGGCGCCAACTCCGGCCGGGACGCCAAGAAGCCGCGGCTGGTGCGCTCCTCCT 414

*TaAFP-D1b* GCTGCTTCGGCGCCAACTCCGGCCGGGACGCCAAGAAGCCGCGGCTGGTGCGCTCCTCCT 414

AB360913 CCCTCGCCGCCATGTGCTCGCTCCCGGGCACCAGCGACGACATCGCCGCCGCGACGCCCC 1500

*TaAFP-D1a* CCCTCGCCGCCATGTGCTCGCTCCCGGGCACCAGCGACGACATCGCCGCCGCGACGCCCC 474

*TaAFP-D1b* CCCTCGCCGCCATGTGCTCGCTCCCGGGCACCAGCGACGACATCGCCGCCGCGACGCCCC 474

AB360913 CGCCGGCGCCGCTGATGCGCACCAGCTCGCTCCCCACCGAGACCGAGGAGGAGCGGTGGC 1560

*TaAFP-D1a* CGCCGGCGCCGCTGATGCGCACCAGCTCGCTCCCCACCGAGACCGAGGAGGAGCGGTGGC 534

*TaAFP-D1b* CGCCGGCGCCGCTGATGCGCACCAGCTCGCTCCCCACCGAGACCGAGGAGGAGCGGTGGC 534

AB360913 GCCGGCGCGAGATGCAGAGCCTCAAGCGCCTCCAGGCCAAGCGCAAGCGCCTCGAGCGCC 1620

*TaAFP-D1a* GCCGGCGCGAGATGCAGAGCCTCAAGCGCCTCCAGGCCAAGCGCAAGCGCCTCGAGCGCC 594

*TaAFP-D1b* GCCGGCGCGAGATGCAGAGCCTCAAGCGCCTCCAGGCCAAGCGCAAGCGCCTCGAGCGCC 594

AB360913 GCACCTCCATGAACTCCGGCAAGTCCGGCGGCAGCAGCAGCCGGGACGACGCCCAGGAGC 1680

*TaAFP-D1a* GCACCTCCATGAACTCCGGCAAGTCCGGCGGCAGCAGCAGCCGGGACGACGCCCAGGAGC 654

*TaAFP-D1b* GCACCTCCATGAACTCCGGCAAGTCCGGCGGCAGCAGCAGCCGGGACGACGCCCAGGAGC 654

AB360913 CGCTCTACCCCAGCGCGTTCCAGCTCCGCCGCTCCGTCGTCGACCAGGGGAACGCTTCCT 1740

*TaAFP-D1a* CGCTCTACCCCAGCGCGTTCCAGCTCCGCCGCTCCGTCGTCGACCAGGGGAACGCTTCCT 714

*TaAFP-D1b* CGCTCTACCCCAGCGCGTTCCAGCTCCGCCGCTCCGTCGTCGACCAGGGGAACGCTTCCT 714

AB360913 CAAGCATGCCGGAGCAAGGTATACACATGCTTTCACCAGCTTCCCTGCTACTCCAACTAT 1800

*TaAFP-D1a* CAAGCATGCCGGAGCAAGGTATACACATGCTTTCACCAGCTTCCCTGCTACTCCAACTAT 774

*TaAFP-D1b* CAAGCATGCCGGAGCAAGGTATACACATGCTTTCACCAGCTTCCCTGCTACTCCAACTAT 774

AB360913 TTGCTACAATCAGTTTGCAATTCCCACTGTTTTATTGCGCTTCCGTGCTTGAATTATTCA 1860

*TaAFP-D1a* TTGCTACAATCAGTTTGCAATTCCCACTGTTTTATTGCGCTTCCGTGCTTGAATTATTCA 834

*TaAFP-D1b* TTGCTACAATCAGTTTGCAATTCCCACTGTTTTATTGCGCTTCCGTGCTTGAATTATTCA 834

AB360913 TTCAGCTGCCTGTTAAGCTCTCGGCGATCCATGCAGGTCGGTGTCATGCCGAGCTTACCG 1920

*TaAFP-D1a* TTCAGCTGCCTGTTAAGCTCTCGGCGATCCATGCAGGTCGGTGTCATGCCGAGCTTACCG 894

*TaAFP-D1b* TTCAGCTGCCTGTTAAGCTCTCGGCGATCCATGCAGGTCGGTGTCATGCCGAGCTTACCG 894

AB360913 TGGTTCTTTTGGTAGGAGATGCATGGGCAGAGGGGGGATCTAACATGTCGAGTGTTCTTT 1980

*TaAFP-D1a* TGGTTCTTTTGGTAGGAGATGCATGGGCAGAGGGGGGATCTAACATGTCGAGTGTTCTTT 954

*TaAFP-D1b* TGGTTCTTTTGGTAGGAGATGCATGGGCAGAGGGGGGATCTAACATGTCGAGTGTTCTTT 954

AB360913 GCCATGGATCTTGCTTTGGTCTTGTGATTTACTGGGATCGATTCGTTAGAACGCTAGTTG 2040

*TaAFP-D1a* GCCATGGATCTTGCTTTGGTCTTGTGATTTACTGGGATCGATTCGTTAGAACGCTAGTTG 1014

*TaAFP-D1b* GCCATGGATCTTGCTTTG**A**TCTTGTGATTTACTGGGATCGATTCGTTAGAACGCTAGTTG 1014

AB360913 AGCCGATGCTTCTTTTTCGCCAAATTGGCTGACAGCTGCCCGCCATGGAAATGGGGAATT 2100

*TaAFP-D1a* AGCCGATGCTTCTTTTTCGCCAAATTGGCTGACAGCTGCCCGCCATGGAAATGGGGAATT 1074

*TaAFP-D1b* AGCCGATGCTTCTTTTTCGCCAAATTGGCTGACAGCTGCCCGCCATGGAAATGGGGAATT 1074

AB360913 CTTTTCTGCACAATCTGATGATACTTCTACGTACGCATGGGATTTGTTGTGTTCTTTTCG 2160

*TaAFP-D1a* CTTTTCTGCACAATCTGATGATACTTCTACGTACGCATGGGATTTGTTGTGTTCTTTTCG 1134

*TaAFP-D1b* CTTTTCTGCACAATCTGATGATACTTCTACGTACGCATGGGATTTGTTGTGTTCTTTTCG 1134

AB360913 GGGCGTTTGTTTTGGGTGATGTCATTTCTGGGATTATTTCGAGCCGTGCTGTTGCTCCTA 2220

*TaAFP-D1a* GGGCGTTTGTTTTGGGTGATGTCATTTCTGGGATTATTTCGAGCCGTGCTGTTGCTCCTA 1194

*TaAFP-D1b* GGGCGTTTGTTTTGGGTGATGTCATTTCTGGGATTATTTCGAGCCGTGCTGTTGCTCCTA 1194

AB360913 GGGTCTGAAGAGATGCCTTCTACGGCATGTTTCTAAGCAGTTTCTAAGCTTTTATTCACT 2280

*TaAFP-D1a* GGGTCTGAAGAGATGCCTTCTACGGCATGTTTCTAAGCAGTTTCTAAGCTTTTATTCACT 1254

*TaAFP-D1b* GGGTCTGAAGAGATGCCTTCTACGGCATGTT**C**CTAAGCAGTTTCTAAGCTTTTATTCACT 1254

AB360913 ACTACTCCCAAGTTAGTAGAAAGTTGAGTCATCTATTTTGGAACGGAGGGATTAATAGTT 2340

*TaAFP-D1a* ACTACTCCCAAGTTAGTAGAAAGTTGAGTCATCTATTTTGGAACGGAGGGATTAATAGTT 1314

*TaAFP-D1b* ACTACTCCCAAGTTAGTAGAAAGTTGAGTCATCTATTTTGGAACGGA**C**GGATTAATAGTT 1314

AB360913 ACGTAGCCTCAGGGTATGATTATTCAGACTAATCATTATCCGAGCAGCTACCAAATATAT 2400

*TaAFP-D1a* ACGTAGCCTCAGGGTATGATTATTCAGACTAATCATTATCCGAGCAGCTACCAAATATAT 1374

*TaAFP-D1b* ACGTAGCCTCAGGGTATGATTATTCAGACTAATCATTATCCGAGCAGCTACCAAATATAT 1374

AB360913 ACGATTTGAATTGTCTACCTGAAAAACTGGAAAGCACTGACGATTAGTAAACGGAAAAGA 2460

*TaAFP-D1a* ACGATTTGAATTGTCTACCTGAAAAACTGGAAAGCACTGACGATTAGTAAACGGAAAAGA 1434

*TaAFP-D1b* ACGATTTGAATTGTCTACCTGAAAAACTGGAAAGCACTGACGATTAGTAAACGGAAAAGA 1434

AB360913 ACACATTGGGATATGCATCTTTATTGACATGTTGGAATATGAAAATGTGACATCTTCTCC 2520

*TaAFP-D1a* ACACATTGGGATATGCATCTTTATTGACATGTTGGAATATGAAAATGTGACATCTTCTCC 1494

*TaAFP-D1b* ACACATTGGGATATGCATCTTTATTGACATGTTGGAATATGAAAATGTGACATCTTCTCC 1494

AB360913 TCTGCATTGGTGCAGGTAGCGGTGATGGCGCTGAGGCGAAGAGCACATCGAGCATGGAGA 2580

*TaAFP-D1a* **C**CTGCATTGGTGCAGGTAGCGGTGATGGCGCTGAGGCGAAGAGCACATCGAGCATGGAGA 1554

*TaAFP-D1b* TCTGCATTGGTGCAGGTAGCGGTGATGGCGCTGAGGCGAAGAGCACATCGAGCATGGAGA 1554

AB360913 TATCTTCTGATAATAATAACCAGAACAAATCCCTCCCGCCGCCGGCACCATCTACGGCCG 2640

*TaAFP-D1a* TATCTTCTGATAATAATAACCAGAACAAATCCCTCCCGCCGCCGGCACCATCTACGGCCG 1614

*TaAFP-D1b* TATCTTCTGATAATAATAACCAGAACAAATCCCTCCCGCCGCCGGCACCATCTACGGCCG 1614

AB360913 GGAAGCTGCCGAACGGCATCGTCAAGGAGCAACCGCCGTTGCGGACCCTCAGGTCGCTGA 2700

*TaAFP-D1a* GGAAGCTGCCGAACGGCATCGTCAAGGAGCAACCGCCGTTGCGGACCCTCAGGTCGCTGA 1674

*TaAFP-D1b* GGAAGCTGCCGAACGGCATCGTCAAGGAGCAACCGCCGTTGCGGACCCTCAGGTCGCTGA 1674

AB360913 CGATGCGCACGACGAGCACCGGCGACCTGCGGAAGAGCATGATGGAGGACATGCCGATAG 2760

*TaAFP-D1a* CGATGCGCACGACGAGCACCGGCGACCTGCGGAAGAGCATGATGGAGGACATGCCGAT**G**G 1734

*TaAFP-D1b* CGATGCGCACGACGAGCACCGGCGACCTGCGGAAGAGCATGATGGAGGACATGCCGAT**G**G 1734

AB360913 TCTCCTCCAAGGTGGACGGCCCCAAC**A**GCAAGAAGATCGACGGCTTCCTCTACAAGTACA 2820

*TaAFP-D1a* TCTCCTCCAAGGTGGACGGCCCCAACGGCAAGAAGATCGACGGCTTCCTCTACAAGTACA 1794

*TaAFP-D1b* TCTCCTCCAAGGTGGACGGCCCCAACGGCAAGAAGATCGACGGCTTCCTCTACAAGTACA 1794

AB360913 GGAAAGGGGAGGAGGTGAGGATAGTGTGCGTCTGCCACGGCAACTTCCTCACGCCGGCGG 2880

*TaAFP-D1a* GGAAAGGGGAGGAGGTGAGGATAGTGTGCGTCTGCCACGGCAACTTCCTCACGCCGGCGG 1854

*TaAFP-D1b* GGAAAGGGGAGGAGGTGAGGATAGTGTGCGTCTGCCACGGCAACTTCCTCACGCCGGCGG 1854

AB360913 AGTTCGTGAAGCACGCCGGCGGCGGCGACGTCACGAATCCGCTCAGGCACATCGTCGTCA 2940

*TaAFP-D1a* AGTTCGTGAAGCACGCCGGCGGCGGCGACGTCACGAATCCGCTCAGGCACATCGT.............. 1909

*TaAFP-D1b* AGTTCGTGAAGCACGCCGGCGGCGGCGACGTCACGAATCCGCTCAGGCACATCGT.............. 1909

Fig S3. Sequence comparison of two new *TaAFP-D* alleles of *TaAFP-D1a* and *TaAFP-D1b* detected in Chinese germplasm with *TaAFP-D*(AB360913).SNPs are in bold letters
