# Supplementary material for: A 4-bp deletion in the 5’UTR of TaAFP-B is associated with seed dormancy in common wheat (Triticum aestivum L.)
Source: BMC Plant Biol. 2019 Aug 9;19:349. doi: 10.1186/s12870-019-1950-4 (PMC6688260; doi:10.1186/s12870-019-1950-4)
Supplement: Supplementary file 4 — Table S1. Polymorphism of the AFPB marker in 91 white-grained cultivars with different levelsof seed dormancy. (DOCX 20 kb) [file 12870_2019_1950_MOESM4_ESM.docx]

Table S1. Polymorphism of the *AFPB* marker in 91 white-grained cultivars with different levels of seed dormancy

| No | Cultivar | GI  (%) | PCR fragment size (-bp) | No | Cultivar | GI  (%) | PCR fragment size (-bp) |
| --- | --- | --- | --- | --- | --- | --- | --- |
| 1 | Bainong64 | 0.23 | 203 | 47 | Shannong757 | 0.17 | 203 |
| 2 | Baisuibai | 0.25 | 203 | 48 | Shanyou225 | 0.25 | 203 |
| 3 | Baiyingdong2 | 0.65 | 207 | 49 | Shi4185 | 0.43 | 203 |
| 4 | Baiyuhua | 0.39 | 203 | 50 | Shijiazhaung8 | 0.56 | 207 |
| 5 | CA0175 | 0.45 | 203 | 51 | Shixin733 | 0.31 | 203 |
| 6 | CA0178 | 0.24 | 207 | 52 | Suiningtuotuomai | 0.1 | 203 |
| 7 | CA0306 | 0.62 | 203 | 53 | Taishan008 | 0.56 | 207 |
| 8 | CA0420 | 0.49 | 203 | 54 | Taishan9818 | 0.47 | 203 |
| 9 | CA0465 | 0.54 | 203 | 55 | Tuhulutou | 0.09 | 203 |
| 10 | CA0471 | 0.46 | 203 | 56 | Tutoumai(jia) | 0.44 | 203 |
| 11 | CA0481 | 0.37 | 203 | 57 | Waitoubai | 0.07 | 203 |
| 12 | Chuan362 | 0.05 | 203 | 58 | Wanxianbaimaizi | 0.08 | 203 |
| 13 | Fengchan3 | 0.04 | 203 | 59 | Xiaobaiyuhua | 0.04 | 203 |
| 14 | Gaocheng8901 | 0.42 | 207 | 60 | Xiaoyan22 | 0.59 | 207 |
| 15 | Han3475 | 0.37 | 203 | 61 | Xiaoyan54 | 0.19 | 203 |
| 16 | Han5316 | 0.48 | 207 | 62 | Xiaoyan6 | 0.14 | 203 |
| 17 | Han6172 | 0.67 | 207 | 63 | Xiaoyuhua | 0.1 | 203 |
| 18 | Hengguan35 | 0.51 | 203 | 64 | Xinmai11 | 0.36 | 207 |
| 19 | Hengguan9526 | 0.37 | 203 | 65 | Xinmai18 | 0.38 | 203 |
| 20 | Hongliang4 | 0.6 | 203 | 66 | Xinmai9 | 0.47 | 203 |
| 21 | Hongsuibai | 0.51 | 203 | 67 | Xinong88 | 0.42 | 203 |
| 22 | Huaimai20 | 0.43 | 207 | 68 | Xinong979 | 0.33 | 203 |
| 23 | Huaimai8 | 0.38 | 203 | 69 | Xumai856 | 0.42 | 203 |
| 24 | Huixianhong | 0.29 | 203 | 70 | Xuyong | 0.07 | 203 |
| 25 | Jimai19 | 0.58 | 207 | 71 | Yanfu188 | 0.42 | 207 |
| 26 | Jimai21 | 0.18 | 203 | 72 | Yangxiaomai | 0.08 | 203 |
| 27 | Jinan16 | 0.62 | 203 | 73 | Yannong15 | 0.37 | 203 |
| 28 | Jinan17 | 0.4 | 207 | 74 | Yannong19 | 0.54 | 203 |
| 29 | Jinmai5 | 0.47 | 203 | 75 | Yanshi4110 | 0.41 | 203 |
| 30 | Jishi02-1 | 0.52 | 207 | 76 | Yanzhan1 | 0.33 | 203 |
| 31 | Kenong9204 | 0.3 | 207 | 77 | Yibinbaimaizi | 0.24 | 203 |
| 32 | Langzhong  baimaizi | 0.08 | 203 | 78 | Yongchuanbaimaizi | 0.23 | 207 |
| 33 | Liangyu99 | 0.33 | 207 | 79 | Yumai18 | 0.07 | 203 |
| 34 | Linmai2 | 0.33 | 203 | 80 | Yumai2 | 0.51 | 203 |
| 35 | Lumai14 | 0.5 | 203 | 81 | Yumai34 | 0.45 | 203 |
| 36 | Lumai21 | 0.32 | 207 | 82 | Yumai47 | 0.37 | 207 |
| 37 | Neixiang173 | 0.08 | 203 | 83 | Zheng366 | 0.24 | 203 |
| 38 | Neixiang19 | 0.24 | 203 | 84 | Zheng9023 | 0.37 | 203 |
| 39 | Nuerhong | 0.16 | 203 | 85 | Zhengmai004 | 0.51 | 207 |
| 40 | Peilinxuxu  baimai | 0.07 | 203 | 86 | Zhongyu5 | 0.36 | 203 |
| 41 | PH82-2 | 0.29 | 203 | 87 | Zhongyu6 | 0.43 | 203 |
| 42 | Pumai9 | 0.2 | 203 | 88 | Zhou8425B | 0.56 | 207 |
| 43 | Rongchang  baimaizi | 0.1 | 203 | 89 | Zhoumai16 | 0.49 | 207 |
| 44 | Shan160 | 0.37 | 203 | 90 | Zhoumai19 | 0.24 | 203 |
| 45 | Shan213 | 0.49 | 203 | 91 | Zimai12 | 0.4 | 207 |
| 46 | Shan253 | 0.23 | 203 |  |  |  |  |
